# Developmental Programming Mediated by Complementary Roles of Imprinted *Grb10* in Mother and Pup

**DOI:** 10.1371/journal.pbio.1001799

**Published:** 2014-02-25

**Authors:** Michael Cowley, Alastair S. Garfield, Marta Madon-Simon, Marika Charalambous, Richard W. Clarkson, Matthew J. Smalley, Howard Kendrick, Anthony R. Isles, Aled J. Parry, Sara Carney, Rebecca J. Oakey, Lora K. Heisler, Kim Moorwood, Jason B. Wolf, Andrew Ward

**Affiliations:** 1 Department of Biology & Biochemistry and Centre for Regenerative Medicine, University of Bath, Bath, United Kingdom; 2 Department of Medical & Molecular Genetics, King's College London, London, United Kingdom; 3 Department of Pharmacology, University of Cambridge, Cambridge, United Kingdom; 4 Department of Physiology, Development and Neuroscience, University of Cambridge, Cambridge, United Kingdom; 5 Cardiff School of Biosciences, Cardiff University, Cardiff, United Kingdom; 6 European Cancer Stem Cell Research Institute, Cardiff School of Biosciences, Biomedical Sciences Building, Cardiff University, Cardiff, United Kingdom; 7 Behavioural Genetics Group, MRC Centre for Neuropsychiatric Genetics and Genomics, Neuroscience and Mental Health Research Institute, Schools of Medicine and Psychology, Cardiff University, Cardiff, United Kingdom; Institute of Science and Technology Austria (IST Austria), Austria

## Abstract

Developmental programming links growth in early life with health status in adulthood. Although environmental factors such as maternal diet can influence the growth and adult health status of offspring, the genetic influences on this process are poorly understood. Using the mouse as a model, we identify the imprinted gene *Grb10* as a mediator of nutrient supply and demand in the postnatal period. The combined actions of *Grb10* expressed in the mother, controlling supply, and *Grb10* expressed in the offspring, controlling demand, jointly regulate offspring growth. Furthermore, *Grb10* determines the proportions of lean and fat tissue during development, thereby influencing energy homeostasis in the adult. Most strikingly, we show that the development of normal lean/fat proportions depends on the combined effects of *Grb10* expressed in the mother, which has the greater effect on offspring adiposity, and *Grb10* expressed in the offspring, which influences lean mass. These distinct functions of *Grb10* in mother and pup act complementarily, which is consistent with a coadaptation model of imprinting evolution, a model predicted but for which there is limited experimental evidence. In addition, our findings identify *Grb10* as a key genetic component of developmental programming, and highlight the need for a better understanding of mother-offspring interactions at the genetic level in predicting adult disease risk.

## Abstract 2

A mouse genetic study reveals that a single gene acting in both mother and offspring has a central role in the uniquely mammalian phenomenon of nutrient provisioning through the placenta and the mammary gland.

## Author Summary

Experiences during early life can impact on health status in adulthood; low birth weight, for example, is linked to an increased risk of diabetes and obesity in later life. Such developmental programming can be influenced by environmental factors such as diet, but the importance of genetics in this process is not well understood. Using the mouse as a model, we investigate the gene *Grb10*, which is imprinted, meaning that it is expressed from only one of its two copies. We show that *Grb10* is a key mediator of developmental programming, controlling supply and demand of nutrients in the postnatal period and influencing growth and body composition. Specifically, we find that *Grb10* determines the proportions of lean and fat tissue during development, and that this is dependent on the combined actions of *Grb10* in the mother and offspring. Our findings have two main implications. First, they suggest that the functions of *Grb10* in mother and offspring are coadapted, providing support for a coadaptation model for the evolution of imprinted genes. Second, they highlight the need for a better grasp of how maternal and offspring genetics interact during development if we are to understand more fully the causes of complex adult disorders such as obesity.

## Introduction

Growth during prenatal and postnatal development influences adult health status. In humans, low birth weight is associated with an increased risk of metabolic diseases, including obesity and diabetes [1]. In addition to overall size, disproportionate growth during development is also a risk factor for common adult diseases, including coronary heart disease and high blood pressure [2]. It is well-established that environmental stresses, such as poor maternal diet [3], can influence offspring growth, but the genetic control of developmental programming is not fully understood. Additionally, most studies have focused on prenatal development, but development in the postnatal period is also critical in influencing adult health status (reviewed in [4]).

The genetic control of growth and nutrient acquisition *in utero* is mediated in part by imprinted genes, defined by their expression from a single parental allele [5]. In the placenta, paternally expressed genes generally promote growth and maternally expressed genes suppress growth. For many genes, this pattern is thought to reflect conflict between the parental genomes, played out within the offspring, over maternal resource allocation [6]. Females maximise lifetime reproductive success by evenly distributing their resources to offspring (since a mother is equally related to all of her offspring). However, when there is multiple paternity within or among litters, males are only related to some offspring produced by a female, and so it is in his genetic interest for his offspring to extract as much of his mate's resources as possible. The functions of a number of imprinted genes are consistent with the predictions of this “parental conflict hypothesis.” For example, deletion of the paternally expressed *Igf2* P0 transcript in the mouse placenta causes a failure in the supply of nutrients to the developing fetus, resulting in growth restriction [7]. Conversely, deficiency of the maternally expressed gene *Grb10* causes placental overgrowth and increases placental efficiency [8]. Conflict between the parental genomes appears to be conserved in humans [9].

Postnatally, the majority of imprinted genes influence aspects of metabolism or behaviour. *Grb10* is an intriguing example of an imprinted gene with diverse postnatal functions. We have previously generated and characterised *Grb10* knockout mice to show that *Grb10* is expressed from the maternally inherited allele in most peripheral tissues but expression in the central nervous system (CNS) is from the paternally inherited allele [10]. Consistent with these sites of parent-of-origin specific expression, we have demonstrated that maternally expressed *Grb10* controls placental and fetal growth [8],[11], as well as adiposity and energy homeostasis in adulthood [12],[13], while paternally expressed *Grb10* tempers social dominance [10]. This reciprocal imprint, with the parental alleles of *Grb10* influencing distinct physiological and behavioural processes, raises questions about the evolution of imprinting at this and other loci. The presence of imprinted gene expression beyond weaning, i.e., after the cessation of parental provisioning, is not consistent with the original predictions of the parental conflict hypothesis [14]. However, theoretical analyses have demonstrated that there are conditions that can favour imprinted expression later in life, but the expectations for patterns of imprinting in adults are not as clear as those associated with imprinting during the phase of parental care [15],[16]. It is also possible that selection for imprinting at a locus occurs only during development and the imprint simply persists into adulthood.

Postnatal nutrient provisioning by mothers occurs through the mammary gland, but unlike the placenta, the genome of the offspring's father is not represented in this tissue, precluding it as a site of direct parental conflict over allocation of resources to offspring. Despite this, we find that *Grb10* is expressed and imprinted in the mammary epithelium during lactation. We show that *Grb10* controls postnatal nutrient supply through expression of the maternally inherited allele in the lactating mammary gland, but at the same time, expression of the maternally inherited allele in the offspring controls nutrient demand. Proportionate growth as in wild-type (WT) animals requires a combination of *Grb10* expression in the mother, which influences offspring adiposity, and *Grb10* expression in the offspring, which influences lean mass. Together, these findings have two key implications. Firstly, our data suggest that *Grb10* mediates both sides of the mother-offspring interaction. The coordinated pleiotropic effects of the gene suggest a possible role for *Grb10* in mother-offspring coadaptation [17]. Coadaptation has been shown theoretically to potentially favour the evolution of imprinting [18], but there is limited empirical evidence to support this. Secondly, when coupled with our previous observations that *Grb10*-associated control of lean/fat proportions influences adult energy homeostasis, our data identify *Grb10* as a key genetic mediator of developmental programming. Moreover, the need for expression of *Grb10* in both mother and offspring to achieve WT lean/fat proportions suggests that a better understanding of mother-offspring interactions at the genetic level will be required for more accurate prediction of adult disease risk.

## Results

**Figure 1:**
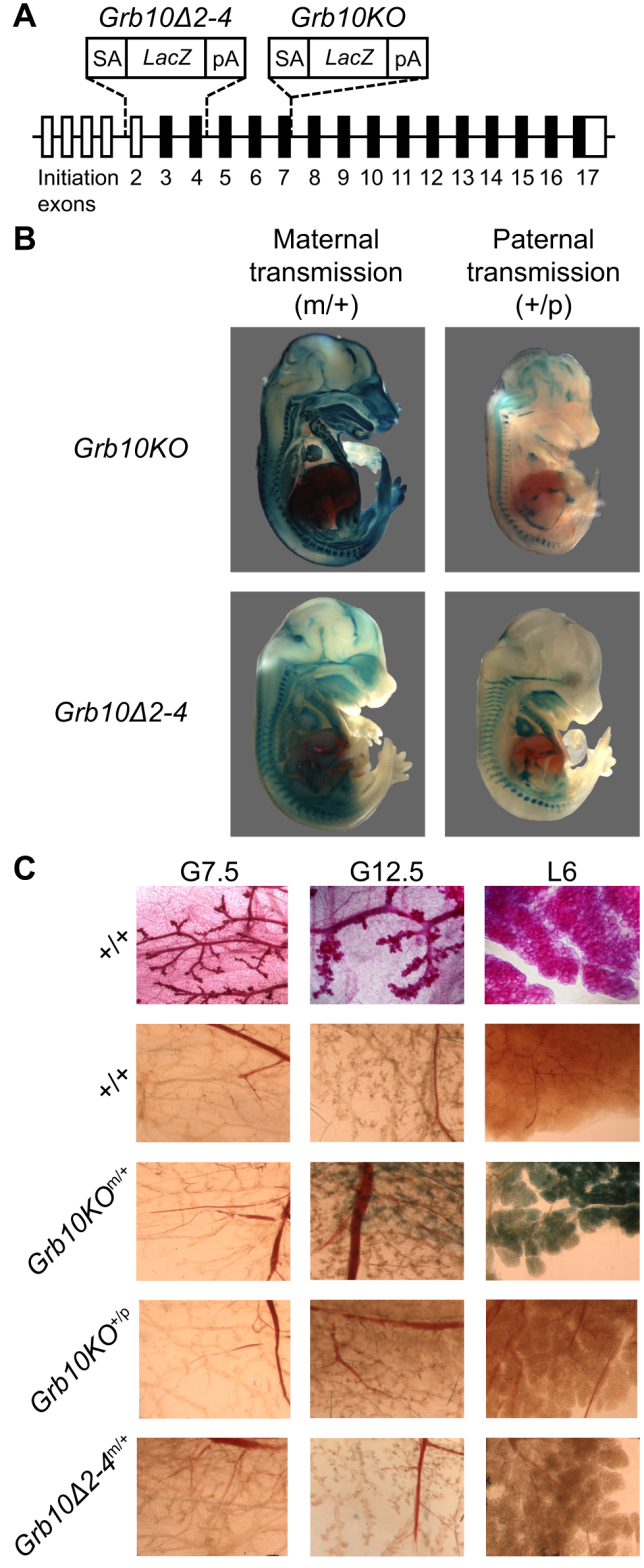
Comparison of *Grb10KO* and *Grb10Δ2-4* mice. (A) Structure of *Grb10*, according to UCSC annotation, showing numbered exons (boxes) and translated regions (filled boxes). The integrated gene-trap cassettes include splice acceptor (SA) and polyadenylation (pA) signals, and a *LacZ* reporter. (B) Comparative *LacZ* staining of bisected embryos at e14.5 inheriting the *Grb10KO* and *Grb10Δ2-4* alleles through each of the parental lines. CNS expression observed in *Grb10KO* ^+/p^ embryos is not detected in *Grb10Δ2-4* ^+/p^ embryos. (C) Comparative *LacZ* staining of adult mammary glands at days 7.5 and 12.5 of gestation (G7.5, G12.5) and day 6 of lactation (L6), showing pregnancy-dependent reporter expression in *Grb10KO* ^m/+^ but not *Grb10Δ2-4* ^m/+^ females. WT (+/+) glands were stained with carmine alum to illustrate morphological changes.

We have previously described two mouse models of *Grb10* ablation, generated by the integration of a *LacZ* reporter gene-trap cassette [10],[11]. Here we more precisely map the gene trap loci (Figures 1A and S1), and also confirm and directly compare the patterns of fetal *LacZ* expression (Figure 1B). Both the *Grb10Δ2-4* and *Grb10KO* alleles ablate Grb10 protein with essentially identical phenotypic consequences, yet expression from their *LacZ* reporter genes is not always equivalent. Maternal transmission of each allele produces similar *LacZ* expression patterns at embryonic day 14.5 (e14.5; Figure 1B). Contrastingly, *LacZ* expression in the CNS is apparent after paternal transmission of the *Grb10KO* allele (*Grb10KO* ^+/p^ embryos) but is not detected in the CNS of *Grb10Δ2-4* ^+/p^ embryos. Expression in adult *Grb10Δ2-4* ^+/p^ brain is detectable but weak relative to *Grb10KO* ^+/p^ (Figure S2).

**Figure 2:**
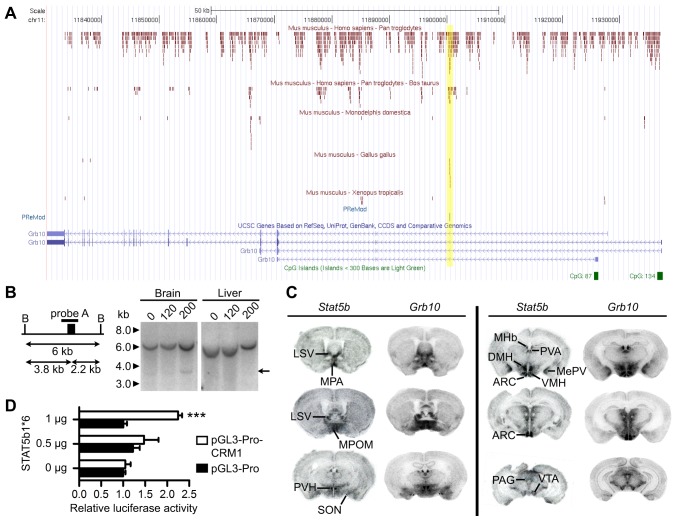
Characterisation of CRM1 and STAT5-mediated expression of *Grb10*. (A) *In silico* identification of conserved elements among selected vertebrate sequences. Conserved intronic sequences between *Grb10* homologs are plotted against annotated mouse transcripts. The PReMod track shows the position of the single regulatory module (CRM1). This site aligns with a sequence highly conserved between mouse, human, chimpanzee, cow, and chicken (highlighted). (B) Assay for DNase I hypersensitivity at CRM1, using probe A. A 6 kb *Bam*HI fragment was detected in all samples. A 3.8 kb DNase I digestion fragment was detected in brain, but not liver, chromatin exposed to 200 U DNase I (arrow). The label “B” indicates a *Bam*HI site. (C) *In situ* hybridisation autoradiographs showing examples of overlapping sites of *Grb10* and *Stat5b* mRNA expression in adult mouse brain, including the arcuate nucleus of the hypothalamus (ARC), dorsomedial nucleus of the hypothalamus (DMH), lateral septal nucleus (LSV), medial amygdaloid nucleus (posteroventral part) (MePV), medial habenular nucleus (MHb), medial preoptic nucleus (MPA), median preoptic nucleus (medial part) (MPOM), periaqueductal grey (PAG), paraventricular thalamic nucleus (PVA), paraventricular nucleus of the hypothalamus (PVH), supraoptic nucleus (SON), ventromedial nucleus of the hypothalamus (VMH), and ventraltegmental area (VTA). (D) *In vitro* transfection assay of the enhancer capability of CRM1. Luciferase activity was measured in cells transfected with a minimal promoter driving luciferase (pGL3-Pro) or with CRM1 cloned upstream of the minimal promoter (pGL3-Pro-CRM1). Only pGL3-Pro-CRM1 responded to increasing doses of constitutively active STAT5b (STAT5b1*6). ***p<0.001 (one-way ANOVA).

Gene-trap cassette integration in the *Grb10Δ2-4* allele is coincident with the deletion of 36 kb of endogenous sequence, while only 12 bp are deleted in *Grb10KO* (Figures 1A and S1) [11]. We considered that the differences in *LacZ* expression between *Grb10KO* ^+/p^ and *Grb10Δ2-4* ^+/p^ brains might be attributed to a tissue-specific enhancer perturbed by cassette integration in the *Grb10Δ2-4* allele. A screen of the *Grb10* genomic sequence for similarity to regulatory elements in Transfac, using PReMod [19], identified a single element of 70 bp, called *cis*-regulatory module 1 (CRM1), that is highly conserved among vertebrates (Figure 2A), and represents a candidate enhancer element.

CRM1 proved hypersensitive to DNase I digestion in mouse adult brain, but not liver where *Grb10* is not transcribed (Figure 2B), consistent with CRM1 being an enhancer. PReMod identified potential recognition sequences within CRM1 for Signal transducer and activator of transcription (STAT)5a/STAT5b, Tst-1, TCF11, and Pax family members (Figure S3A). We reasoned that only factors spatially overlapping with *Grb10* expression could potentially regulate *Grb10 in vivo*, and using public expression data [20], we ruled out all but STAT5b. Using mRNA *in situ* hybridisation, we confirmed that *Stat5b* brain expression overlaps extensively with *Grb10* (Figure 2C). In a cell transfection assay, CRM1 demonstrated enhancer capability in the presence of constitutively active STAT5b (Figure 2D). The STAT5 recognition sequences [21], but not those of Tst-1, TCF11, or Pax, are 100% conserved between mouse, human, chimpanzee, cow, and chicken (Figure S3B). Together, these data suggest STAT5 promotes *Grb10* expression in mouse brain. Although CRM1 is not within the deleted sequence of *Grb10Δ2-4*, its 5′ end is within 366 bp of the deletion. STAT5 binding, or its affect on *Grb10* transcription, might therefore be perturbed by the deletion in the *Grb10Δ2-4* allele, which could account for the observed expression differences in the CNS. One explanation is that the deletion alters the local chromatin conformation, reducing the interactions between CRM1 and the *Grb10* promoter. Consistent with this, the deletion includes at least one binding site in brain for CTCF [22], a regulator of chromatin architecture.

*Grb10* was previously identified as a STAT5-responsive gene in mammary epithelial cells [24]. More recently, genome-wide ChIP-seq mapping of STAT5 binding sites in mammary tissue identified three binding sites within the Grb10 locus, including one coincident with CRM1, confirming that CRM1 can bind STAT5 in vivo [23]. We examined *Grb10* expression in the mammary epithelia of our mouse models utilising *LacZ* reporter activity as a readout, predicting that reporter expression from the *Grb10Δ2-4* allele would be weaker than from the *Grb10KO* allele because of a perturbation of CRM1 activity, similar to the differences observed in the CNS. We first demonstrated pregnancy-dependent expression of *Grb10* in mammary epithelium using *Grb10KO* ^m/+^ females. No reporter activity was detected at day 7.5 of gestation (G7.5), a subset of epithelial cells were *LacZ*-positive at G12.5, and widespread epithelial expression was observed at day 6 of lactation (Figure 1C), an expression profile comparable with other transcriptional targets of STAT5 signalling [24]. Mammary epithelial expression is restricted to the maternally inherited copy (no *LacZ* staining was detected in *Grb10KO* ^+/p^ females), consistent with *Grb10* imprinting in other peripheral tissues; expression of the *Grb10* maternal allele is widespread during fetal development [8],[10],[11] and in neonatal tissues (Figure S4), but more restricted in the adult [10],[12]. Comparable with expression differences in the CNS, no *LacZ* expression was detected in *Grb10Δ2-4* ^m/+^ epithelium.

**Figure 3:**
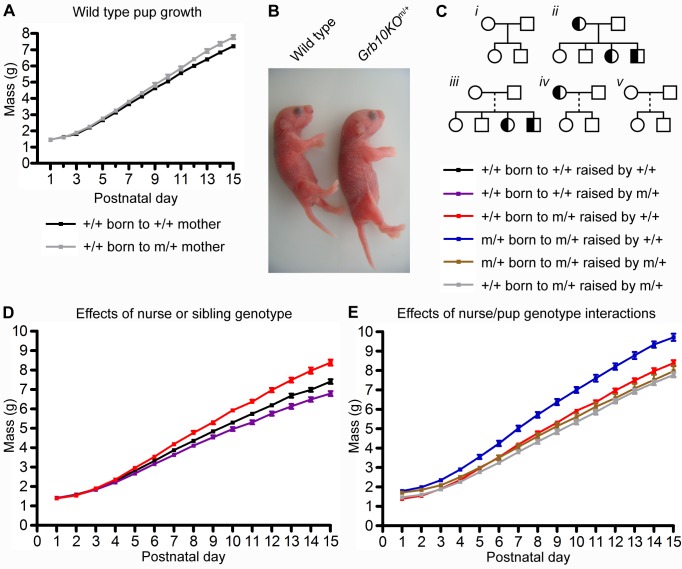
*Grb10* controls postnatal supply and demand. (A) WT (+/+) pups born to *Grb10KO* ^m/+^ (m/+) dams gained more weight to postnatal day 15 than WT pups born to WT dams. (B) *Grb10KO* ^m/+^ and WT male siblings on the day of birth. (C) Schematic of dam/pup relationships studied. All crosses used WT sires (white squares). WT dams (white circles) gave birth only to WT pups (*i*), while *Grb10KO* ^m/+^ dams (half-filled circles) gave birth to mixed litters of WT and *Grb10KO* ^m/+^ pups (*ii*). Cross-fostering (dashed line) enabled switching of dam/pup genotypes (*iii* and *iv*). Pure WT litters were cross-fostered to non-biological WT nurses as a control (*v*). (D) Effects of nurse or sibling genotype on WT pup growth. (E) Effects of *Grb10* genotype interactions between nurse and offspring on pup growth. Values represent means ± standard error.

The functional significance of imprinting in the mammary gland, which regulates nutrient allocation in the postnatal period, has not been widely considered. We were therefore intrigued by the pregnancy-dependent, imprinted expression of *Grb10* observed in the mammary gland and its potential functional importance. Pup growth is the ultimate correlate of gland function [25], and we thus compared growth of WT (+/+) pups born to WT and *Grb10KO* ^m/+^ dams. At e17.5, WT embryos of *Grb10KO* ^m/+^ females are 10% smaller than those of WT females, due to an increased litter size [8]. Despite this embryonic growth disadvantage, WT pups born to *Grb10KO* ^m/+^ dams gained more weight postnatally than those born to WT dams, after standardising litter size (Figure 3A). We initially interpreted this as an enhanced provisioning capacity of *Grb10KO* ^m/+^ dams, suggesting that *Grb10* functions in mothers to suppress nutrient supply postnatally. However, WT pups born to *Grb10KO* ^m/+^ dams also had *Grb10KO* ^m/+^ siblings, which were 16%±5.4% larger than their WT littermates at birth (Figures 3B, 3C, and S5A). It was therefore necessary to consider whether these larger siblings might be impacting on WT growth, and to separate as far as possible these effects from the genotype of the dam.

We used a cross-fostering strategy to differentiate the postnatal growth effects of *Grb10* ablation in pup from those in dam and sibling (Figure 3C). Similar approaches have been used previously to differentiate between parental and offspring effects on traits, including in the context of genomic imprinting (e.g., [26]–[29]). Pup weights at days 1, 8, and 15 were modelled as described in Materials and Methods. Data were analysed using a generalised linear mixed model (GLMM). Cross-fostering had no main effect on any trait (*p*>0.2 for all traits; Table S1), consistent with other studies, and therefore was not included in the final models (see also Figure S5B). The effect of *Grb10* ablation in the dam alone was assessed by comparing the growth of pure WT litters raised by WT or *Grb10KO* ^m/+^ nurses. Reduced weight gain was observed in pups raised by *Grb10KO* ^m/+^ nurses (Figure 3D, purple line) compared to those raised by WT nurses (black), demonstrating that *Grb10KO* ^m/+^ nurses exhibit compromised nutrient supply, and therefore the function of *Grb10* in the mother is to promote nutrient provisioning postnatally. Compromised supply from *Grb10KO* ^m/+^ nurses was confirmed by our modelling, which showed a significant effect of nurse genotype at day 8 (F_1,21_ = 15.08; *p*<0.001) and day 15 (F_1,21.6_ = 25.60; *p*<0.0001), but not day 1 (F_1,19.9_ = 0.53; *p* = 0.4744) (Table 1, which presents hypotheses tested and key findings from the data; Tables S1, S2, S3, which present the results of the models in full). The observations in our initial experiment that WT pups born to *Grb10KO* ^m/+^ dams gained more weight postnatally than those born to WT dams could therefore not be attributed to dam genotype alone, but were likely to be influenced by sibling genotype. In support of this, WT pups with *Grb10KO* ^m/+^ siblings gained more weight than WT pups with only WT siblings, when raised by WT nurses (Figure 3D, red and black, respectively). This is consistent with the idea that *Grb10KO* ^m/+^ pups exhibit increased demand for nutrients, to which WT nurses respond with improved provisioning, enabling increased weight gain in WT littermates. However, because all litters born to *Grb10KO* ^m/+^ dams contained at least one *Grb10KO* ^m/+^ pup, this result is also consistent with a maternal effect in which exposure to the *Grb10KO* ^m/+^ uterine environment promotes increased postnatal weight gain in both WT and *Grb10KO* ^m/+^ pups. Although the effects of siblings and the dam are correlated, we examined whether the postnatal growth of an individual is influenced by the frequency of *Grb10KO* ^m/+^ pups in their litter. This analysis supported the conclusions that the pattern of postnatal growth reflects a demand effect, with postnatal growth of a pup increasing as a function of the frequency of *Grb10KO* ^m/+^ siblings, with this relationship being significant for growth from day 1 to day 8 (*β* = 0.97, 21 degrees of freedom [df], *p* = 0.022) and from day 1 to day 15 (*β* = 1.34, 21.5 df, *p* = 0.018). Thus, *Grb10* has pleiotropic and complementary roles in dam and pup, enhancing nutrient supply in dams and suppressing demand in pups.

**Table 1:** Summary of hypotheses tested by the datasets obtained in the cross-fostering study.

Hypothesis	Comparison (Source)	Supporting Evidence *p*-Value from General Linear Model			Interpretation
		Day 1	Day 8	Day 15	
The process of cross-fostering does not influence pup growth	Fostered vs. non-fostered pups^a^	*p* = 0.22	*p* = 0.24	*p* = 0.73	No significant effect from cross-fostering, therefore this effect was excluded from the final models
m/+ nurses have reduced nutrient supply	Size of pups raised by +/+ vs. m/+ nurses^b^	*p* = 0.47	*p* = 0.0009	*p*<0.0001	Pups gain more weight if suckling from +/+ nurses than if suckling from m/+ nurses
m/+ pups have increased nutrient demand	+/+ pups vs. m/+ pups^b^	*p*<0.0001	*p*<0.0001	*p* = 0.0002	m/+ pups are larger than +/+ sibs at days 1, 8, and 15
+/+ nurses are more responsive to high m/+ pup demand than m/+ nurses	m/+ pups raised by +/+ nurses vs. m/+ pups raised by m/+ nurses^c^	*p* = 0.086	*p*<0.0001	*p*<0.0001	m/+ pups gain more weight if suckling from +/+ nurses than if suckling from m/+ nurses
*Grb10* ablation in both nurse and pup normalises m/+ pup overgrowth to weight of +/+ pups suckling from +/+ nurses	+/+ pups raised by +/+ nurses vs. m/+ pups raised by m/+ nurses^c^	*p*<0.0001	*p* = 0.53	*p* = 0.084	m/+ pups suckling from m/+ nurses are not significantly different from +/+ pups suckling from +/+ nurses at days 8 and 15, despite being larger at day 1

Pup weights on day 1, day 8, and day 15 of the study period were compared using GLMM to determine the effects of pup genotype, biological dam genotype, and nurse genotype. Each row states the hypothesis to be tested, the relevant comparisons to be made to test the hypothesis, *p*-values at the three time points determined from the models, and an interpretation of the results from the GLMM. Comparisons are those shown graphically in Figure 3. Details of the model are presented in the Materials and Methods. The “source” in column 2 refers to one of Tables S1, S2, S3 that contains the full model results.

a Table S1.

b Table S2.

c Table S3.

We next considered the compound effects of *Grb10* ablation in nurse and pup, hypothesising that more demanding *Grb10KO* ^m/+^ pups might not reach their full size potential when suckling from *Grb10KO* ^m/+^ nurses with reduced supply. Supporting this, *Grb10KO* ^m/+^ pups raised by WT nurses gained more weight than those raised by *Grb10KO* ^m/+^ nurses during the postnatal period, despite being similar in weight at day 1 (Figure 3E, blue and brown, respectively). Modelling the data confirmed significant differences in weight between *Grb10KO* ^m/+^ pups raised by WT and *Grb10KO* ^m/+^ nurses at day 8 (t = 4.78, 43.6 df, *p*<0.0001) and day 15 (t = 5.47, 52.3 df, *p*<0.0001), but not at day 1 (t = 1.76, 43.7 df, *p* = 0.086) (Tables 1 and S2). *Grb10KO* ^m/+^ pups suckling from *Grb10KO* ^m/+^ nurses were larger than their WT littermates at day 1, but their growth trajectories converged within a few days (Figure 3E, brown and grey, respectively). However, *Grb10KO* ^m/+^ pups remained larger than WT siblings throughout the experimental period when suckling from WT nurses (Figure 3E, blue and red, respectively). Modelling of the pup/nurse interaction confirmed these observations, with the contrast between *Grb10KO* ^m/+^ pups suckling from WT nurses compared to other combinations being significant at day 8 (F_1,62.3_ = 39.33; *p*<0.0001) and day 15 (F_1,70.4_ = 38.83; *p*<0.0001), while the difference between *Grb10KO* ^m/+^ pups with *Grb10KO* ^m/+^ nurses was significantly different to that of WT pups with *Grb10KO* ^m/+^ nurses at day 1 (t = 6.28, 113 df, *p*<0.0001), but not day 8 (t = 1.48, 115 df, *p* = 0.14) or day 15 (t = 0.25, 119 df, *p* = 0.80) (Table S3). Thus, oversized *Grb10KO* ^m/+^ neonates rapidly adjust to WT size after birth, but only when the nurse genotype is also *Grb10KO* ^m/+^, implying a role for *Grb10* in influencing mother-offspring coadaptation. Our cross-fostering experiments show that WT body size is achieved through the complementary actions of *Grb10* in mother and offspring.

**Figure 4:**
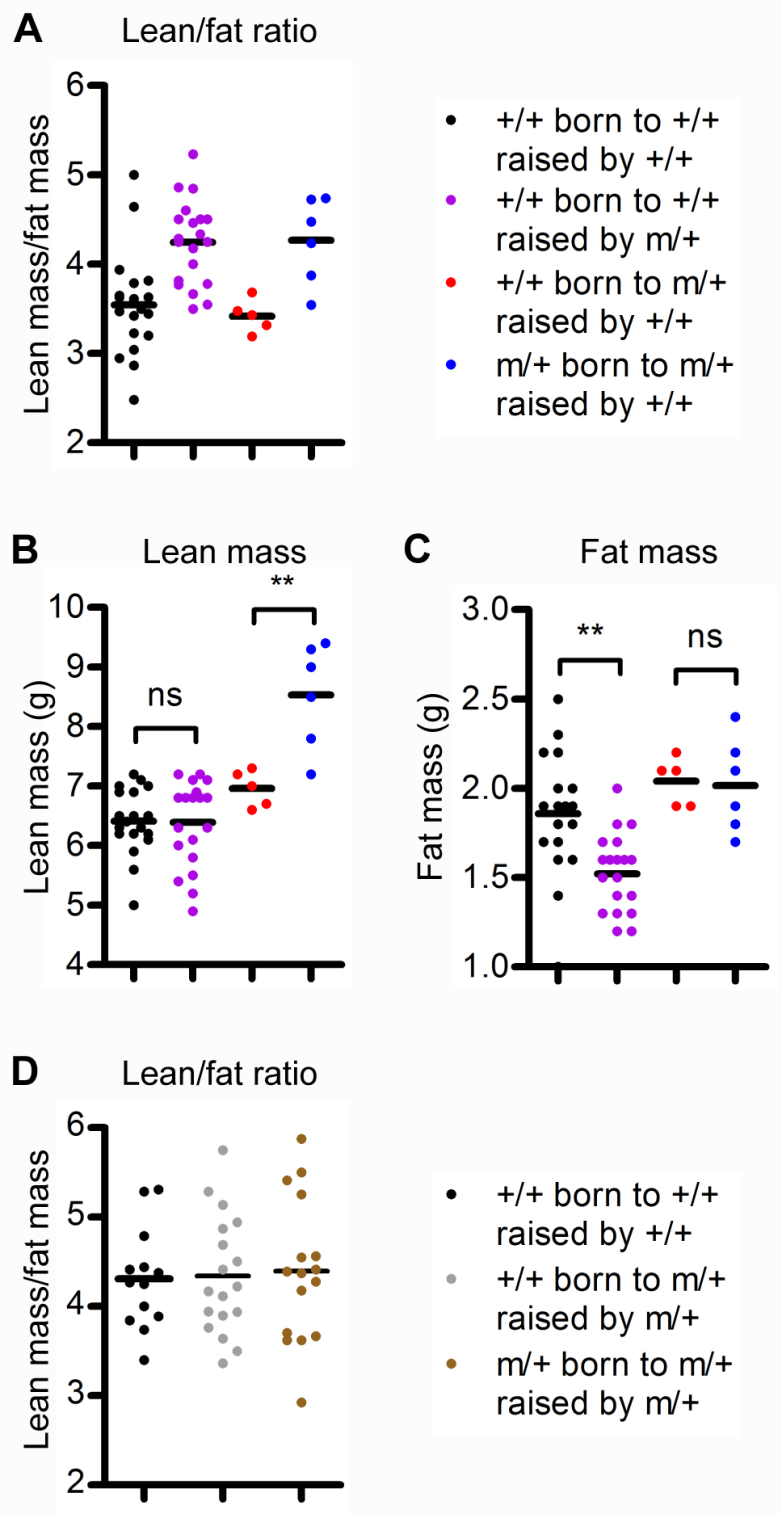
Functional *Grb10* is required in mother and pup for WT offspring body proportions. (A) Lean/fat mass ratio in a subset of cross-fostered pups indicating that *Grb10* ablation in either nurse or pup increases the lean/fat mass ratio relative to WT pups raised by WT nurses. (B) Total lean mass. (C) Total fat mass. (D) Lean/fat mass ratio in a subset of cross-fostered pups, indicating that the body composition of *Grb10KO* ^m/+^ pups raised by *Grb10KO* ^m/+^ nurses is similar to that of WT pups raised by WT or *Grb10KO* ^m/+^ nurses. Data points represent individual animals; mean values are represented by horizontal lines. Datasets in (B) and (C) were analysed using one-way ANOVA with Tukey's post hoc test. **p<0.01. ns, not significant.

In addition to overall size, disproportionate growth can also be a risk factor for adult disease. In adulthood, *Grb10Δ2-4* ^m/+^ and *Grb10KO* ^m/+^ mice have an altered body composition, exhibiting increased lean mass and reduced adiposity relative to WT animals, which results in enhanced glucose metabolism [12],[13]. The finding in the present study that growth is influenced by *Grb10* in both mother and pup prompted us to examine the lean/fat ratios of animals used in the cross-fostering study. More specifically, we set out to ask whether the increased lean/fat ratio of a *Grb10KO* ^m/+^ mouse is a result of *Grb10* depletion within that mouse, or if the genotype of the nurse also contributes to this phenotype. To address this question, we analysed the body composition of cross-fostered pups at the end of the growth study period using dual-emission X-ray absorptiometry (DXA). Ablating *Grb10* in either nurse or pup increased the lean/fat ratio compared to WT pups raised by WT nurses (Figure 4A). When *Grb10* was ablated in offspring alone, the increased lean/fat ratio was caused by a gain in lean mass, with fat mass unchanged (Figure 4B and 4C; compare red with blue). Conversely, *Grb10* ablation in nurse alone caused a reduction in adipose tissue, while lean mass remained unchanged (Figure 4B and 4C; compare black with purple). Therefore, *Grb10* expressed in the mother has the major influence on adipose deposition, while offspring *Grb10* largely influences lean mass. A WT lean/fat ratio requires functional *Grb10* in both mother and pup.

Since *Grb10KO* ^m/+^ neonates adjust to WT size when suckling from a *Grb10KO* ^m/+^ nurse, we asked whether this normalisation effect was also reflected in the lean/fat ratios. Consistent with the adjustment in body size, the lean/fat ratio of *Grb10KO* ^m/+^ animals raised by *Grb10KO* ^m/+^ nurses was comparable to that of their WT littermates, and also to that of WT pups raised by WT nurses (Figure 4D; compare brown with grey and black). This provides further evidence that the pleiotropic functions of *Grb10* in mother and pup are complementary.

To inform on the mechanism through which *Grb10* regulates postnatal nutrient supply, we examined *Grb10KO* ^m/+^ mammary gland gross morphology at different stages, but did not detect any obvious differences from WT glands (Figure S6A). We also measured various parameters in histological sections of glands harvested at day 5 of lactation. Since we had shown that *Grb10KO* ^m/+^ pups demonstrate increased nutrient demand, we compared glands from *Grb10KO* ^m/+^ dams to glands from *Grb10KO* ^+/p^ dams. Unlike WT dams, both *Grb10KO* ^m/+^ and *Grb10KO* ^+/p^ dams raise comparable mixed genotype litters, but the absence of *Grb10* expression from the paternally-expressed allele in mammary glands (Figure 1C) means that *Grb10KO* ^+/p^ females are effectively WT for *Grb10* in this tissue. No differences were observed in total abdominal gland weight or surface area (Figure S6B and S6C). To gain more detailed insight into gland structure, we quantified the total number, total area, mean area, mean perimeter, mean Feret diameter and mean minimum Feret diameter for lumina and adipocytes, but found no differences (Figures S6D and S6E). These analyses were repeated on a separate cohort of glands isolated 48 hours after a forced wean at day 15 of lactation, and we made similar observations (unpublished data). In support of these morphometric analyses, no significant differences were found between WT and *Grb10KO* ^m/+^ glands, in immunofluorescence experiments using antibodies to markers of luminal epithelial (cytokeratin-18 [CK18]) and myoepithelial (CK14) cells (Figure S7A and S7B). Moreover, fluorescence activated cell sorting (FACS) showed no differences in the number or proportions of the same key cell types (Figure S7C and S7D).

As a measure of milk letdown from dam to pups, pup weight gain was assessed following a period of separation from the dam. We found no evidence that the reduced nutrient provisioning of *Grb10KO* ^m/+^ dams observed in our earlier experiments was due to compromised milk letdown since, if anything, they were able to transfer more milk to pups than *Grb10KO* ^+/p^ dams (Figure S8A). Although *Grb10* is almost exclusively expressed from the paternally inherited allele in adult brain, and therefore *Grb10KO* ^m/+^ females are unlikely to demonstrate perturbed maternal behaviour, we confirmed that pup retrieval and nest building behaviours are comparable between *Grb10KO* ^m/+^ and *Grb10KO* ^+/p^ dams (Figure S8B–S8E). The protein and fat content of milk was also comparable between nurses used in the cross-fostering study (Figure S9A and S9B). Together these data suggest that milk letdown, maternal behaviour or the proportions of fat and protein in milk are not the basis for reduced provisioning in *Grb10KO* ^m/+^ dams. However, prolactin expression in the pituitary glands of *Grb10KO* ^m/+^ nurses was significantly elevated relative to WT nurses, when raising WT litters, whereas pituitary growth hormone levels were unchanged (Figure S9C). Reduced provisioning causes pups to suckle more vigorously, promoting maternal pituitary prolactin expression that normally stimulates increased milk production [30]. Our data suggest that mammary glands of *Grb10KO* ^m/+^ females are resistant to elevated prolactin.

## Discussion

In mice, most imprinted genes are expressed in the placenta and many have been shown experimentally to influence placental development and function. The parental conflict hypothesis, which, at a gross level, predicts that paternally expressed genes promote growth while maternally expressed genes suppress growth, is consistent with the functions of several genes imprinted in the placenta, including *Grb10* [8],[11]. While the conflict hypothesis can potentially explain the occurrence of imprinting at many loci, the functions of a considerable number of imprinted genes cannot be easily reconciled with the predictions of the model, such as those involved in maternal care behaviours. Recent extensions to the model have considered cases in which asymmetries between genes inherited from mothers and fathers can arise from various patterns of interactions with kin, but they do not provide strong predictions about the nature of imprinting in adult tissues [15],[31]. There are also alternative models that do not consider conflict, such as the maternal-offspring coadaptation model, which describes how the combination of alleles expressed in mothers and their offspring jointly determines offspring fitness [18]. Whether each of these models could potentially account for the complex patterns of *Grb10* expression and imprinting is unclear, but we present here some of the strongest empirical evidence that coadaptation could play a role, without necessarily ruling out alternative hypotheses.

Earlier studies involving reciprocal crosses, and cross-fostering, between two distinct mouse strains established that parent-of-origin effects such as genomic imprinting could contribute to coadaptation between genotypes (e.g., [26],[29]). These studies provide evidence that maternal provisioning is influenced by maternal and offspring genotypes [27] and that provisioning is optimal when mother and offspring are of the same genotype [29]. A study mapping quantitative trait loci on adult mouse body weight and organ weights indicated that a number of imprinted loci had small but detectable effects on these traits [28]. This genome-wide mapping is complementary to our approach, the manipulation of a single imprinted gene, which shows that *Grb10* can contribute functionally to body weight and proportions through actions in both mother and offspring.

**Figure 5:**
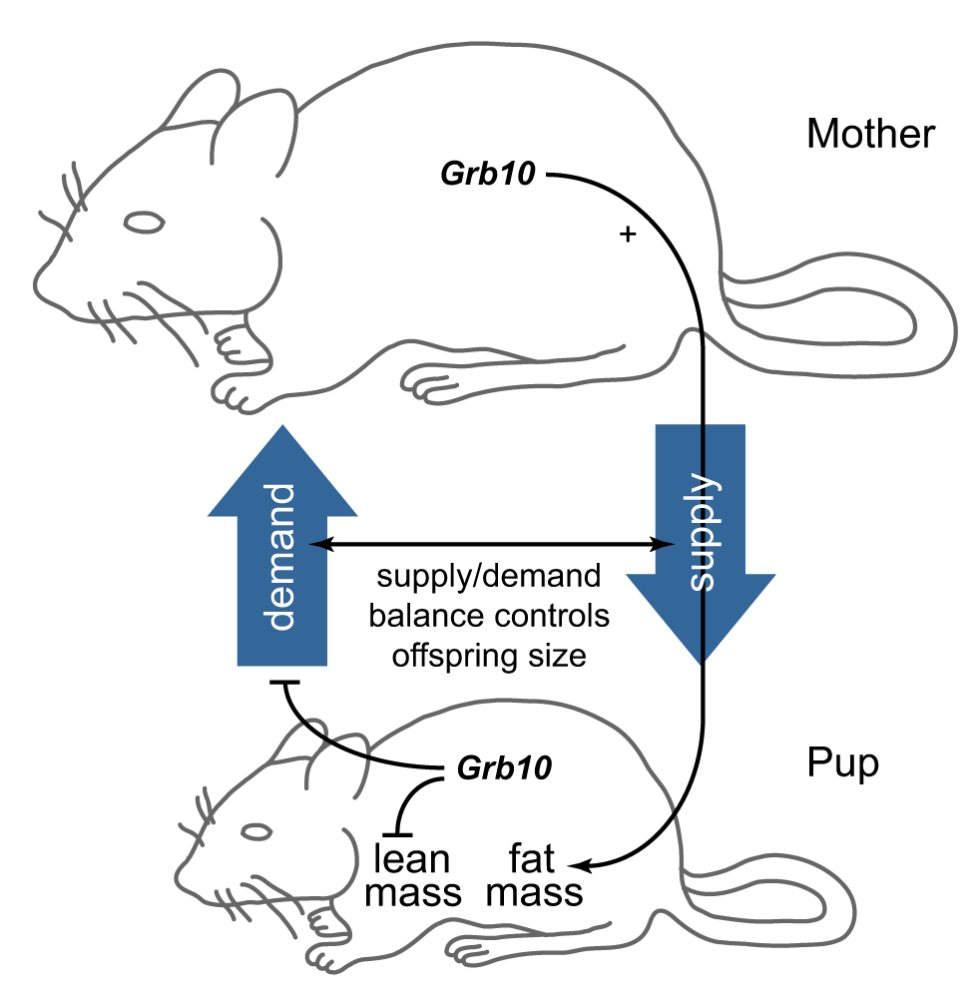
Overview of complementary *Grb10* functions in mother and pup. *Grb10* expressed in the mother promotes postnatal nutrient supply through the mammary gland, while offspring *Grb10* suppresses nutrient demand. Together, this regulation of nutrient acquisition ensures offspring achieve an optimal body size. Body proportions are also influenced by both *Grb10* expressed in the mother and in the offspring. Offspring *Grb10* suppresses the development of lean mass, while offspring fat mass is promoted by *Grb10* expressed in the mother and acting on postnatal nutrient supply, jointly promoting optimal offspring body proportions.

*Grb10* is an intriguing model with which to study imprinted gene function and evolution, because its two parental alleles are expressed in different tissues where they influence distinct physiological and behavioural processes [10]. Our earlier work characterising the same knockout mice used in this study established that maternally expressed *Grb10* regulates fetal and placental growth [8],[11], consistent with the conflict hypothesis, as well as glucose homeostasis in adulthood [12]. In the present study, we demonstrate that *Grb10* also controls postnatal growth through imprinted expression in mammary epithelium. The two archetypal mammalian tissues differ fundamentally in that the placenta contains both maternal and paternal genetic contributions, as in the offspring, but the mammary gland shares only maternal genes with offspring. Thus, while they are functionally analogous in supporting offspring growth, the placenta can be a site of direct conflict between the maternal and paternal genomes, whereas the mammary gland is not [14]. Consequently, our findings do not appear to fit with the simple predictions of the conflict hypothesis. The finding that WT body size and proportions require the combined and complementary actions of *Grb10* in mother and pup (Figure 5), provides support for the coadaptation model of imprinting evolution, although further work would be needed to confirm that the effects of *Grb10* lead to increased fitness. However, even if coadaptation explains the imprinting of *Grb10* in pups, the coadaptation process would not favour imprinted expression in the mammary gland. Either a different hypothesis is needed to explain why the gene is imprinted in this tissue, or the pattern of expression in the mammary gland could simply reflect the selection that led to imprinting earlier in life. It should also be noted that our study compares WT *Grb10* with a single knockout allele. Strong support for the coadaptation theory would come from an analysis of different allelic variants. Pups from combinations where mothers and offspring are expressing the same allelic variant would be expected to have higher fitness than those from combinations expressing different alleles.

Conflict between the parental genomes, coadaptation of processes regulating postnatal nutrient acquisition, or indeed some other driving force, could have provided the initial selective pressure for the evolution of imprinting. However, a mechanism for imprinted gene regulation, once evolved, could facilitate development of novel gene functions, and thus the two models for the evolution of imprinting at the *Grb10* locus, conflict and coadaptation, need not be mutually exclusive [32]. Indeed, expression of both the maternally and paternally inherited alleles of *Grb10* in different tissues, influencing different phenotypes, strongly supports this notion, since the same selective forces presumably could not have led to both patterns of expression. Evidence for the later evolutionary acquisition of novel functions or imprinted expression of a gene in adult tissues could be viewed as consistent with this idea. In this context it is interesting that in at least one marsupial species, the tammar wallaby, *Grb10* is expressed in a range of fetal and adult tissues, including lactating mammary gland, but its expression appears not to be imprinted [33]. Future work on the expression and functions of *Grb10* in non-mammalian species will help to unravel the likely evolutionary course of this pleiotropic gene.

The mechanism through which *Grb10* regulates postnatal nutrient supply is not clear, but its pregnancy-dependent expression in the mammary epithelium is consistent with *Grb10* controlling supply through this tissue. Our data suggest that *Grb10* might mediate the response of the epithelium to pituitary prolactin, but further work will be needed to define its mode of action. We could not detect any phenotype associated with *Grb10KO* in analyses of mammary glands at the cellular level or in analyses of milk fat and protein content. One intriguing possibility is that *Grb10* in mammary epithelium could regulate the release of signalling molecules that influence growth or metabolism of suckling offspring. Evidence exists for such lactocrine signalling playing a role in offspring development [34].

Environmental influences on the programming of adult health status during development have been widely investigated. In humans, maternal and child undernutrition are risk factors for high glucose concentration and blood pressure in adulthood [3]. Chemical insults can also adversely affect development with implications for adult health. Bisphenol A (BPA), which mimics oestrogen, has garnered much attention recently because of its widespread use in the manufacture of baby bottles, coatings for food cans and other commonly used items. Effects of exposure during development to environmentally relevant levels of BPA, using rodent models, include increased body weight, advanced puberty, and altered reproductive function, as well as a possible predisposition to mammary and prostate cancers [35]. This is effected, at least in part, through alterations to the fetal epigenome, including reduced levels of DNA methylation, which is associated with changes in gene transcription [36],[37].

The genetic control of developmental programming of adult health status is relatively poorly understood. We have previously shown that *Grb10* determines the proportions of lean and fat tissue during development, and that altering the lean/fat ratio by ablating *Grb10* affects glucose homeostasis in adulthood [12]. In the present study, we find that this programming of adult metabolic state during postnatal development is not the result of *Grb10* acting in the pup alone, but is achieved through the combined actions of *Grb10* in mother and offspring. Interestingly, two recent mouse studies of nutrient restriction during gestation have demonstrated up-regulation of *Grb10* expression in offspring, consistent with our findings that *Grb10* plays a key role in responding to nutrient supply [38],[39]. Our work highlights the need for a much better understanding of genetic interactions between mother and offspring in predicting adult health status.

## Materials and Methods

### Ethics Statement

Experiments involving mice were conducted under a UK Home Office licence granted following local ethical review.

### *LacZ* Expression and *In Situ* Hybridisation

Embryos were isolated at e14.5, bisected and assayed for β-galactosidase activity as described previously [10]. Abdominal mammary glands (gland number 4) were isolated from gravid and lactating females, all 10 weeks old and virgins at mating. For lactating females, litters were standardised to seven pups at birth. Glands were mounted on APTS-subbed slides, fixed for 2 hours in 2% (w/v) paraformaldehyde (PFA), 0.25% (v/v) glutaraldehyde, 0.01% (v/v) Igepal CA-630 in 0.1× PBS, and further fixed for 2 hours in 2 mM MgCl_2_, 0.01% (w/v) sodium deoxycholate, 0.02% (v/v) Igepal CA-630 in 0.1× PBS. Glands were incubated in X-gal as described for embryos for 18 hours at 28°C. Glands were cleared in acetone for 6.5 hours, dehydrated through an ascending ethanol series and stored in xylene. *In situ* hybridisations and *LacZ* expression analyses performed on adult brain sections have been described previously [10]. The *Stat5b* probe was amplified using forward (5′-GCAAGCATTGTCATTGTCTCCG-3′) and reverse (5′-CCATTCCTACCACCTAATCCTCAG-3′) primers. The *Grb10* probe has been described elsewhere [10].

### Enhancer Analyses

ExactPlus [40] was used to detect sequence conservation among *Grb10* homologs, obtained from the UCSC Genome Browser. The minimum length of exact match to seed was 6 bp; the minimum number of species to seed was equivalent to the number for that alignment; and the minimum number of species to extend a hit was 2. Murine *Grb10* exons were excluded from the alignment. 15 bp of intronic sequence flanking each exon were also excluded to eliminate conserved splice recognition sequences. Custom tracks were submitted to the UCSC Genome Browser. The genomic sequence of murine *Grb10* was submitted to PReMod [19] for analysis.

CRM1 was assessed for DNase I hypersensitivity in adult brain and liver, using a standard method [41] with 0, 120 and 200 units of DNase I. A 799 bp genomic DNA probe A was amplified using forward (5′-GGGTGTTTGTCCTTGATGCT-3′) and reverse (5′-CTGACCCCCAGAATGTGTTT-3′) primers, and radiolabelled using [α-^32^P]dCTP and a Roche High Prime labelling kit.

Probe A was cloned into the *Kpn*I and *Sac*I restriction enzyme sites of the pGL3-Promoter vector (pGL3-Pro, Promega), to generate pGL3-Pro-CRM1. The constitutive STAT5b expression construct, pRSV-puroSTAT5b1*6, has been used elsewhere [42]. Transfections were performed on ∼60% confluent NIH/3T3 cells in six-well plates, using Lipofectamine 2000 (Invitrogen). Cells were co-transfected with 0.1 µg pRL-SV40 (Promega), encoding *Renilla* luciferase. 48 hours after transfection, luciferase activity was quantified using the Dual-Luciferase Reporter Assay System (Promega) and a Microlumat Plus luminometer (EG&G Berthold). Activity levels for each well were normalised to that of *Renilla* luciferase.

### Growth Studies

*Grb10Δ2-4* and *Grb10KO* mice were generated and maintained as described previously on a mixed C57BL6:CBA strain background [10],[11]. WT mice of the same genetic background were used as controls. 7-week-old virgin dams were mated with WT sires and removed to separate cages following the observation of a cervical plug. At birth, pup paws were tattooed to permit identification. Litters were standardised to five to seven pups at birth by arbitrary pup selection. Where appropriate, pups were cross-fostered at birth to nurses that had given birth on the same day. Pups were weighed daily. Figure 3A: +/+ born to +/+, *n* = 20 pups (three litters); +/+ born to m/+, *n* = 18 pups (six litters). Figure 3D/3E: +/+ born to +/+ raised by +/+, *n* = 31 pups (five litters); +/+ born to +/+ raised by m/+, *n* = 27 pups (four litters); +/+ born to m/+ raised by +/+, *n* = 9 pups (four litters); +/+ born to m/+ raised by m/+, *n* = 18 pups (six litters); m/+ born to m/+ raised by +/+, *n* = 12 pups (four litters); m/+ born to m/+ raised by m/+, *n* = 14 pups (six litters). Figure S5B: +/+ raised by biological +/+ dam, *n* = 20 pups (three litters); +/+ raised by nurse +/+ dam, *n* = 31 pups (five litters). Pups were culled on postnatal days 15 or 17, and genotyped following tissue biopsy [11].

### DXA

A subset of animals from the growth studies were analysed by dual-emission X-ray absorptiometry (DXA) (PIXImus scanner, Lunar) [12]. For Figure 4A–4C, animals were analysed at day 17: +/+ born to +/+ raised by +/+, *n* = 19 pups (three litters); +/+ born to +/+ raised by m/+, *n* = 20 pups (three litters); +/+ born to m/+ raised by +/+, *n* = 5 pups (two litters); m/+ born to m/+ raised by +/+, *n* = 6 pups (two litters). For Figure 4D, animals were analysed at day 15: +/+ born to +/+ raised by +/+, *n* = 13 pups from two litters; +/+ born to m/+ raised by m/+, *n* = 18 pups from six litters; m/+ born to m/+ raised by m/+, *n* = 16 pups from six litters.

### General Linear Models

Pup weight was modelled using restricted maximum likelihood in the Mixed Procedure in SAS (SAS Institute) with pup, biological dam, nurse, and the interaction between pup and nurse genotypes fitted as categorical fixed effects and a litter ID as a random effect. Degrees of freedom were determined using the Kenward-Roger Degrees of Freedom Approximation [43], which, in this model, effectively corrects the denominator degrees of freedom for the random effects to avoid pseudoreplication when using replicates sampled from the same litters. The influence of *Grb10KO* ^m/+^ siblings on individual growth was determined by adding a regression variable to the model described above that accounts for the frequency of *Grb10KO* ^m/+^ among the siblings of that individual (so it is measured on the rest of the litter not including the genotype of the focal individual being considered; i.e., it is the frequency that an individual pup experiences within their litter).

### Mammary Gland Morphology

Abdominal glands for carmine alum staining were fixed in Carnoy's fixative (60% ethanol, 30% chloroform, 10% acetic acid) for 3 hours, transferred to 70% ethanol for 15 minutes, and hydrated slowly. Glands were stained in carmine alum (0.2% w/v) carmine dye, 0.5% (w/v) aluminium potassium sulphate) for 18 hours, dehydrated, and stored as above. Abdominal (number 4) glands for morphometric analyses were isolated from *Grb10KO* ^m/+^ and *Grb10KO* ^+/p^ females at day 5 of lactation, all 8–10 weeks old at conception and previously unmated. Note that these dams are the same as those used in the behavioural and milk letdown experiments. Wet weights were recorded and glands were spread across APTS-subbed slides, photographed on grids and surface areas measured using ImageJ. Glands were fixed overnight in 4% PFA (w/v) in PBS, dehydrated and sectioned at a thickness of 8 µm. Sections were stained with haematoxylin and eosin, and photographed at 200× magnification. Measurements were made using ImageJ. The combined measurements from three fields per sample were used. *Grb10KO* ^m/+^, *n* = 4; *Grb10KO* ^+/p^, *n* = 3.

### Gene-Trap Cassette Mapping

Southern blots and PCR were performed using standard protocols. Primer sequences are available upon request.

### Allele-Specific Expression of *Grb10*

RNA isolation from day 1 neonatal tissues, cDNA synthesis, PCR, and sequencing were performed as described previously [44]. 30 cycles of amplification were used with forward (5′-GCTGGACTCTGGTGGAACAC-3′) and reverse (5′-GGCACACATACAGCTTCTTCC-3′) primers.

### Immunofluorescence

WT and *Grb10KO* ^m/+^ abdominal glands were isolated from females 48 hours after a forced wean at day 15 of lactation. All females were 7 weeks old and virgins at mating. The litter size of all females was normalised to 7 pups on the day of birth. Glands were sectioned and fixed as described for haematoxylin and eosin staining. Immunofluorescence was performed essentially as described [45], using the following antibodies: CK14 (LL002, Abcam), CK18 (Ks 18.04, Progen Biotechnik), Alexa555-conjugated goat anti-mouse IgG1 cross-adsorbed (Invitrogen), and Alexa488-conjugated goat anti-mouse IgG3 cross-adsorbed (Invitrogen). Sections were counter-stained with DAPI. Images were taken using a Zeiss LSM510META confocal laser scanning microscope. Cells were counted by assigning DAPI-stained nuclei as either luminal epithelial- (CK18+) or myoepithelial-associated (CK14+). Counts from three fields were combined for each sample. WT, *n* = 3; *Grb10KO* ^m/+^, *n* = 3.

### Flow Cytometry

Single cells were prepared from fourth mammary fat pads of mice aged 17–25 weeks, at day 7–10 of gestation, with pregnancy confirmed during harvest of mammary tissue [46],[47]. Cell suspensions at 10^6^ cells/ml were stained with anti-CD24-FITC (clone M/69 at 1.0 µg/ml; BD Biosciences), anti-Sca-1-APC (clone D7 at 1.0 µg/ml; eBioscience), anti-CD45-PE-Cy7 (clone 30-F11 at 1.0 µg/ml; BD Biosciences), and anti-CD49f-PE-Cy5 (clone GoH3 at 5.0 µl/ml; BD Biosciences). Cells were sorted at low pressure (20 psi using a 100 µm nozzle) on a FACSAria (Becton Dickenson) equipped with violet (404 nm), blue (488 nm), green (532 nm), yellow (561 nm), and red (635 nm) lasers and using FACSDiva software. There was no intervening culture period between cell isolation, staining, and flow sorting. Mammary epithelial cell subpopulations were defined as in [47].

### Milk and Pituitary Gland Analyses

At postnatal day 17, nurses used in the cross-fostering study were anaesthetised with an intraperitoneal injection of 0.1% (w/v) xylazine, 0.5% (w/v) ketaset in 0.9% (w/v) NaCl at 16 µl/g mouse. Milk collection was aided by an intraperitoneal injection of 200 µl 10 IU/ml oxytocin (Sigma Aldrich) in 0.1% PBS. A vacuum pump was used to harvest 100–200 µl milk. Milk fat and protein content were analysed as described [48]. After fat removal, protein was diluted 1/5 in 50 mM Tris HCl (pH 8.0), 150 mM NaCl, 1% (v/v) Igepal CA-630, boiled for 10 minutes, and mixed with an equal volume of reducing sample buffer. Samples were run on a 15% Criterion Tris-HCl gel (Bio-Rad Laboratories) alongside pre-stained molecular weight markers. Fixation and drying were performed using standard methods.

After milk collection, nurses were humanely killed by cervical dislocation and pituitary glands harvested. RNA was extracted from pituitaries using TRI Reagent (Sigma Aldrich). Total RNA (1 µg) was DNase-treated with RQ1 RNase-free DNase I (Promega). cDNA was synthesised using random hexamers with Superscript III RNase H^−^ Reverse Transcriptase (Invitrogen). Real-time PCR (qRT-PCR) was used to measure expression of prolactin and growth hormone normalised to β-actin. Reactions were performed in duplicate and analysed as described previously [8].

### Maternal Behaviour and Milk Letdown

*Grb10KO* ^+/p^ dams were considered to be a better control than WT dams in these experiments because both *Grb10KO* ^m/+^ and *Grb10KO* ^+/p^ dams have mixed genotype litters, and therefore demand is more equally matched than with WT dams that have only WT litters. Nurturing behaviour of dams was quantified, including time to initiate nest building activity, time to settle on the nest, and time to retrieve pups, following removal of pups from the nest and separation from the dam for 1 hour (essentially as in [49], but performed at 3–4 hours into the light cycle on postnatal day 1). As a surrogate measure of milk letdown, pups were weighed immediately following removal from the dam, immediately before their return to the nest, and at intervals for up to four hours thereafter [49].

## Supporting Information

Figure S1 **Mapping of the gene-trap cassette integration sites in** ***Grb10KO*** **and** ***Grb10Δ2-4*** **.** (A) Schematic of the *Grb10* gene around exons 6–8 (black boxes). 5′ RACE experiments (unpublished) demonstrated splicing of transcripts from *Grb10* exon 6 onto the gene-trap cassette in the *Grb10KO* allele, and so cassette integration was initially assumed to be within intron 6. The positions of relevant restriction enzyme recognition sequences and probe B (thick black line) are shown. The amplicons generated by primer sets A–C are illustrated by grey lines. The gene-trap cassette, consisting of a 5′ splice acceptor sequence (SA), *LacZ* reporter, and 3′ polyadenylation sequence (pA), is shown with dashed lines indicating its integration site at the 3′ end of exon 7. A broken line indicates intronic sequence not shown. Diagram not to scale. (B) Upper panel: Southern blots of *Bam*HI- and *Pst*I-digested genomic DNA from tail clips of a WT (+/+) animal and animals heterozygous (+/p) and homozygous (m/p) for the *Grb10KO* allele, challenged with probe B. A 4.5 kb *Bam*HI digestion fragment was detected for all genotypes indicating that cassette integration occurred downstream of the intron 6 *Bam*HI restriction site. A 6.7 kb *Pst*I fragment was detected in WT and *Grb10KO* ^+/p^ DNA, but was absent from *Grb10KO* ^m/p^ DNA. A band of ∼10 kb was detected in DNA from animals heterozygous and homozygous for the *Grb10KO* allele. The gene-trap cassette contains a *Pst*I site ∼4.3 kb from the 5′ end, suggesting the integration site is ∼5.7 kb downstream of the exon 5 *Pst*I site, close to the 3′ end of exon 7. Lower panel: Primer sets A, B, and C were designed to span the approximate site of cassette integration determined by Southern blotting, to enable finer mapping. PCR was performed on WT and *Grb10KO* ^m/p^ DNA. Primer set B failed to amplify from *Grb10KO* ^m/p^ DNA indicating the site of integration was between the two primers. (C) The forward primer from set B was used with a reverse primer complementary to a sequence within the gene-trap cassette to amplify from *Grb10KO* ^m/p^ DNA. The amplicon was cloned and sequenced. Cassette integration (dashed lines) is coincident with the deletion of 11 bp from the 3′ end of exon 7 (bold) and 1 bp from the 5′ end of intron 7. Despite integrating at the 3′ end of exon 7, transcripts initiating upstream splice from exon 6 onto the SA sequence of the gene-trap cassette, as determined by 5′ RACE (unpublished). (D) For *Grb10Δ2-4*, the approximate site of integration has been mapped previously by Southern blotting and is coincident with the deletion of ∼36 kb of endogenous *Grb10* sequence including exons 2–4 [11]. In the present study, the 5′ end of the deleted sequence was mapped with greater resolution using PCR. A schematic of the *Grb10* gene around exon 2 (black box) is shown. Amplicons generated by primer sets D–H are illustrated by grey lines. CRM1 is indicated by a grey box. The gene-trap cassette integration site is illustrated by dashed lines; the 3′ end of the integration site is downstream of exon 4 (unpublished). (E) Primer sets D, E, F, and G were designed to span the approximate site of cassette integration in *Grb10Δ2-4* determined by Southern blotting (not shown). Primer sets D and E readily amplified from WT and *Grb10Δ2-4* ^m/p^ DNA, indicating that the sequence spanned by these primers is present in both genotypes. Primer sets F and G amplified from WT, but not *Grb10Δ2-4* ^m/p^ DNA, indicating that the 5′ end of the deletion lies within the sequence spanned by primer set F. (F) Genomic sequence around CRM1 (bold text) showing its proximity to the 5′ end of the deleted sequence in the *Grb10Δ2-4* allele. The positions of primer sets F and H are indicated. Primer set H amplified from WT and *Grb10Δ2-4* ^m/p^ DNA (not shown) indicating the 5′ end of the deleted sequence lies between the reverse primer of set H (H rev) and the reverse primer of set F (F rev). Genome coordinates are based on mouse build mm9, and were obtained from the UCSC genome browser.
(TIF)

Figure S2 **Comparative** ***LacZ*** **expression in** ***Grb10KO*** **^+/p^ and** ***Grb10Δ2-4*** **^+/p^ adult brain sections.** Adult brain sections were assayed for β-galactosidase activity for the same duration. At all sites, reporter expression was weaker in *Grb10Δ2-4* ^+/p^ than *Grb10KO* ^+/p^ sections. (A)/(A′) Expression in the dorsal raphe nucleus (DRN), ventrotegmental nucleus (VTg), and raphe magnus nucleus (MnR). (B)/(B′) Expression in the ventrotegmental area (VTA) and the substantia nigra pars compacta (SNC) but not the substantia nigra pars reticulata (SNR). (C)/(C′) Expression in the rostral and lateral interpeduncular nucleus (IPR and IPL, respectively) but not the caudal portion (IPC).
(TIF)

Figure S3 **Sequence analysis of CRM1.** (A) The mouse *Grb10* sequence was submitted to PReMod for analysis. A single putative regulatory module of 70 bp (CRM1) was detected, consisting of a number of potential transcription factor binding sites, ranked by relative likelihood scores, calculated by an alignment with consensus sequences in the TransFac 7.2 database. TransFac module codes are presented with transcription factor names on the left hand side. (B) Sequence alignment of CRM1 between four mammalian and one avian species. Potential transcription factor binding sites identified by PReMod are illustrated with brackets. The conserved STAT5 recognition sequences are in bold text.
(TIF)

Figure S4 **Maintenance of** ***Grb10*** **imprinting in neonatal tissues.** Tissues were isolated from day 1 neonates generated from intercrosses of C57Bl6 (B) and *Mus musculus castaneus* (C) animals. cDNA was synthesised and amplified using primers spanning a single nucleotide polymorphism (SNP) in exon 8 between the two parental strains. Amplicons were sequenced across the SNP to determine the parental origin of the expressed allele. For all tissues examined, neonates generated from a B×C cross (maternal genotype presented first) demonstrated expression exclusively from the maternally inherited allele. This finding was reproduced in tissue samples from the reciprocal cross (C×B), confirming that *Grb10* is maternally expressed in postnatal heart, liver, lung, and tongue.
(TIF)

Figure S5 **Additional postnatal growth data.** (A) Growth of WT (+/+) and *Grb10KO* ^m/+^ pups raised by their biological dams. The graph is reproduced from Figure 3A with the addition of the *Grb10KO* ^m/+^ siblings of WT pups born to *Grb10KO* ^m/+^ dams (brown line in Figure 3E), illustrating the postnatal catch-up growth of WT siblings in a mixed litter. (B) Growth of WT pups raised by biological WT and nurse WT mothers, as a control for the process of cross-fostering. No weight differences were detected on the day of birth or on the day after cross-fostering (day 2), at which point any effects of pup rejection would be detectable. No differences were detected at the end of the experimental period (day 15). These data confirm that the process of cross-fostering does not influence pup growth. Modelling of the data also confirmed no effect of cross-fostering at days 1, 8, and 15.
(TIF)

Figure S6 **Assessment of mammary gland morphology in** ***Grb10KO*** **^m/+^ females.** (A) No gross morphological differences were detected between the abdominal mammary glands of WT and *Grb10KO* ^m/+^ dams at G7.5, G12.5, and L6 (WT gland images are reproduced from Figure 1C): ad, adipose tissue; teb, terminal end bud; pr, primary branch; se, secondary branch. (B–E) Abdominal mammary glands were isolated at day 5 of lactation from females raising six of their own pups (no cross-fostering). Glands from *Grb10KO* ^m/+^ females were compared with those from *Grb10KO* ^+/p^ females, which do not show any perturbation of *Grb10* expression in the mammary gland. No significant differences were found for total gland weight (B) or surface area (C), by Student's *t*-test. Sectioned glands were assessed for occupancy, size and shape of lumina (D) and adipocytes (E). No significant differences were found using Student's *t*-test. Similar experiments were also performed on glands isolated 48 hours after a forced wean, but no statistically significant differences were observed (unpublished).
(TIF)

Figure S7 **Cellular analyses of** ***Grb10KO*** **^m/+^ mammary glands.** (A) Representative immunofluorescence image of a WT mammary gland section assessed for luminal epithelial and myopeithelial cell occupancy with anti-CK18 and anti-CK14, respectively. (B) No significant differences were detected between WT and *Grb10KO* ^m/+^ glands for luminal epithelial or myoepithelial cell number, or for the ratio of luminal∶myopepithelial cells, assessed by Student's *t*-test. (C) Epithelial cell population sizes determined by FACS. Representative scatter plots of unstained control samples and samples from mid-gestation WT and *Grb10KO* ^m/+^ dams stained with antibodies to CD24, Sca-1, and CD49f. Only data from the epithelial cells are shown. Non-epithelial cells were gated out prior to this analysis as described [47]. (D) Percentages of myoepithelial, luminal estrogen receptor negative (ER−), and luminal ER+ cells from two independent analyses of mid-gestation WT and *Grb10KO* ^m/+^ mice as gated in (C). Note that the dot plots in (C) correspond to experiment 1.
(TIF)

Figure S8 **Maternal behaviour and milk letdown in** ***Grb10KO*** **^m/+^ females.** Behaviour and letdown in *Grb10KO* ^m/+^ dams was compared to *Grb10KO* ^+/p^ dams on the day of birth following separation of dam and pups for one hour. (A) Mean pup weight change following separation and reunion, as a measure of milk letdown. Letdown appeared to be greater in *Grb10KO* ^m/+^ than *Grb10KO* ^+/p^ dams, and this was significant by Student's *t*-test at 1 hour and 3 hours after reunion. (B–E) Upon reunion, pups were placed in a corner of the cage away from the nest. No significant differences were observed in the time taken for the dam to retrieve the first (B) or last (C) pup, or in the time taken to begin nest building (D) or settle on the nest (E). Datasets were compared with Student's *t*-test. *p<0.05.
(TIF)

Figure S9 **Analyses of nurses used in the cross-fostering study.** (A) Milk was isolated from nurses at day 17 of lactation and analysed for protein content by SDS-PAGE. Each lane represents milk protein isolated from a separate dam. No consistent differences were observed between any of the datasets. The molecular weights (MW) of protein standards are indicated, and bands corresponding to some key milk proteins are annotated: WAP, whey acidic protein. (B) Percentage fat content of milk samples was determined. No statistically significant differences were observed between any dataset, as assessed by one-way ANOVA. Bars represent the mean ± standard error. For +/+ nurse raising a +/+ litter, *n* = 4; for m/+ nurse raising a +/+ litter, *n* = 3; for +/+ nurse raising a mixed litter, *n* = 2. (**C**) Prolactin and growth hormone expression in the pituitary glands of some nurses used in the cross-fostering study, assayed by qPCR and normalised to the expression of β-actin. Prolactin expression was elevated in the glands of *Grb10KO* ^m/+^ nurses suckling WT litters, relative to WT nurse controls. No differences were observed between WT nurses raising pure WT or mixed litters. Expression of growth hormone did not differ significantly between any dataset. *p<0.05, using Kruskal-Wallis test with Dunn's multiple comparison post test.
(TIF)

Table S1 **Tests of cross-fostering effect.** The cross-fostering effect was tested using the same GLMM described in the Materials and Methods. The F tests all have one numerator degree of freedom, so the df column indicates denominator degrees of freedom.
(DOC)

Table S2 **Pup weight modelled at days 1, 8, and 15.** Pup weight was modelled as described in Materials and Methods. Num DF, numerator degrees of freedom. Den DF, denominator degrees of freedom. +/+, WT. m/+, *Grb10KO* ^m/+^. Model estimated means were generated using the Least-Squares Means statement in the Mixed Procedure in SAS from the full model.
(DOCX)

Table S3 **Pairwise tests of differences in pup weights at days 1, 8, and 15 as a function of the genotype of nurse and pup.** Rows and columns indicate the pup/nurse genotype combinations being compared. m/+ indicates the *Grb10KO* ^m/+^ genotype while +/+ indicates the WT genotype. d is the difference in means between the two categories being compared (calculated as the mean of the value indicated by the row combination minus the column combination). The estimated model means being compared are given in Table S2.
(DOC)

## Abbreviations

CK: cytokeratin
CNS: central nervous system
CRM1: *cis*-regulatory module 1
df: degrees of freedom
e14.5: embryonic day 14.5
FACS: fluorescence activated cell sorting
GLMM: generalised linear mixed model
STAT: Signal transducer and activator of transcription
WT: wild type
