# Supplementary figures and images for: Developmental Programming Mediated by Complementary Roles of Imprinted Grb10 in Mother and Pup
Source: PLoS Biol. 2014 Feb 25;12(2):e1001799. doi: 10.1371/journal.pbio.1001799 (PMC3934836; doi:10.1371/journal.pbio.1001799)

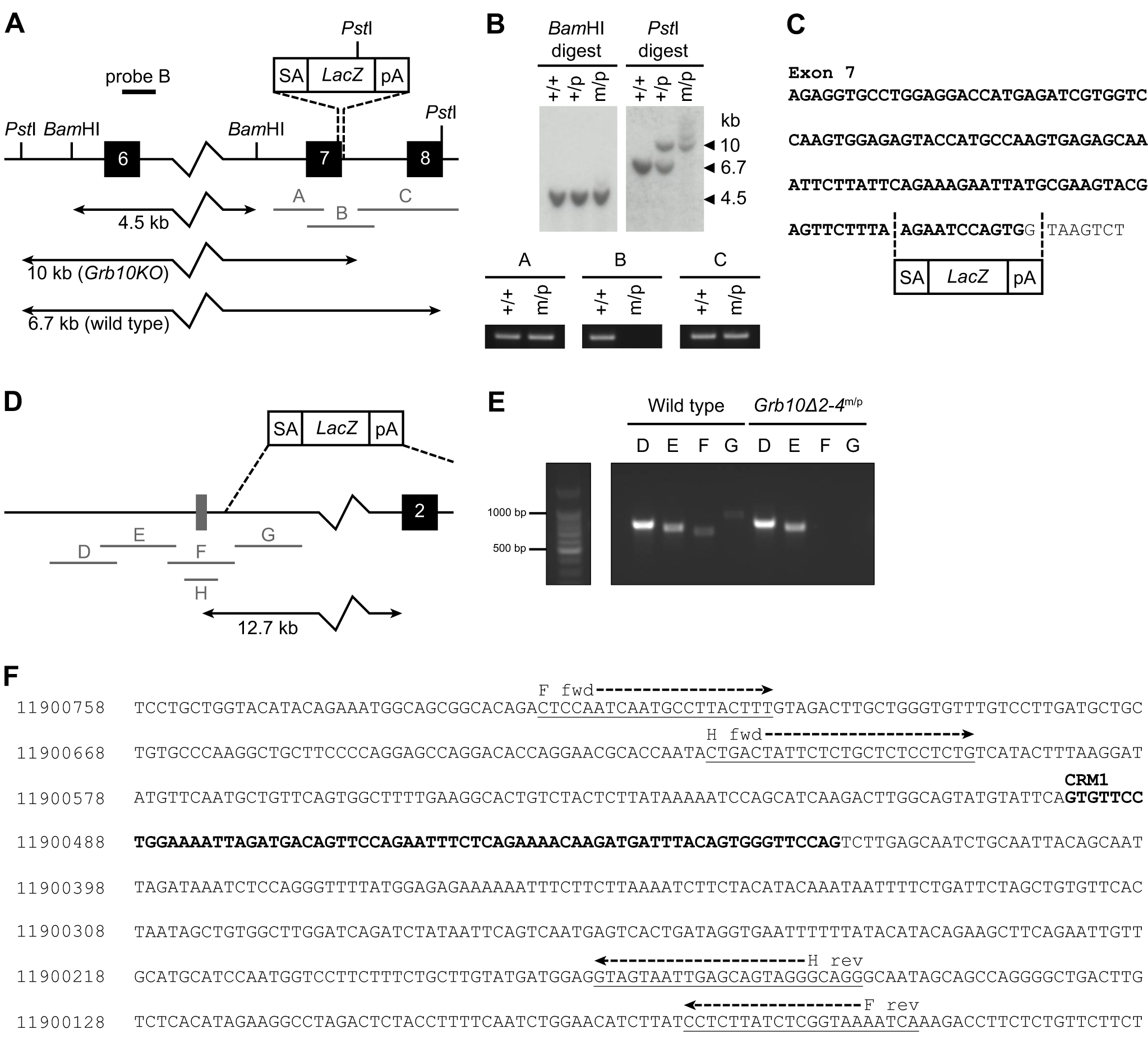

Supplement: Figure S1 — Mapping of the gene-trap cassette integration sites in Grb10KO and Grb10Δ2-4 . (A) Schematic of the Grb10 gene around exons 6–8 (black boxes). 5′ RACE experiments (unpublished) demonstrated splicing of transcripts from Grb10 exon 6 onto the gene-trap cassette in the Grb10KO allele, and so cassette integration was initially assumed to be within intron 6. The positions of relevant restriction enzyme recognition sequences and probe B (thick black line) are shown. The amplicons generated by primer sets A–C are illustrated by grey lines. The gene-trap cassette, consisting of a 5′ splice acceptor sequence (SA), LacZ reporter, and 3′ polyadenylation sequence (pA), is shown with dashed lines indicating its integration site at the 3′ end of exon 7. A broken line indicates intronic sequence not shown. Diagram not to scale. (B) Upper panel: Southern blots of BamHI- and PstI-digested genomic DNA from tail clips of a WT (+/+) animal and animals heterozygous (+/p) and homozygous (m/p) for the Grb10KO allele, challenged with probe B. A 4.5 kb BamHI digestion fragment was detected for all genotypes indicating that cassette integration occurred downstream of the intron 6 BamHI restriction site. A 6.7 kb PstI fragment was detected in WT and Grb10KO +/p DNA, but was absent from Grb10KO m/p DNA. A band of ∼10 kb was detected in DNA from animals heterozygous and homozygous for the Grb10KO allele. The gene-trap cassette contains a PstI site ∼4.3 kb from the 5′ end, suggesting the integration site is ∼5.7 kb downstream of the exon 5 PstI site, close to the 3′ end of exon 7. Lower panel: Primer sets A, B, and C were designed to span the approximate site of cassette integration determined by Southern blotting, to enable finer mapping. PCR was performed on WT and Grb10KO m/p DNA. Primer set B failed to amplify from Grb10KO m/p DNA indicating the site of integration was between the two primers. (C) The forward primer from set B was used with a reverse primer complementary to a sequence within [file pbio.1001799.s001.tif]

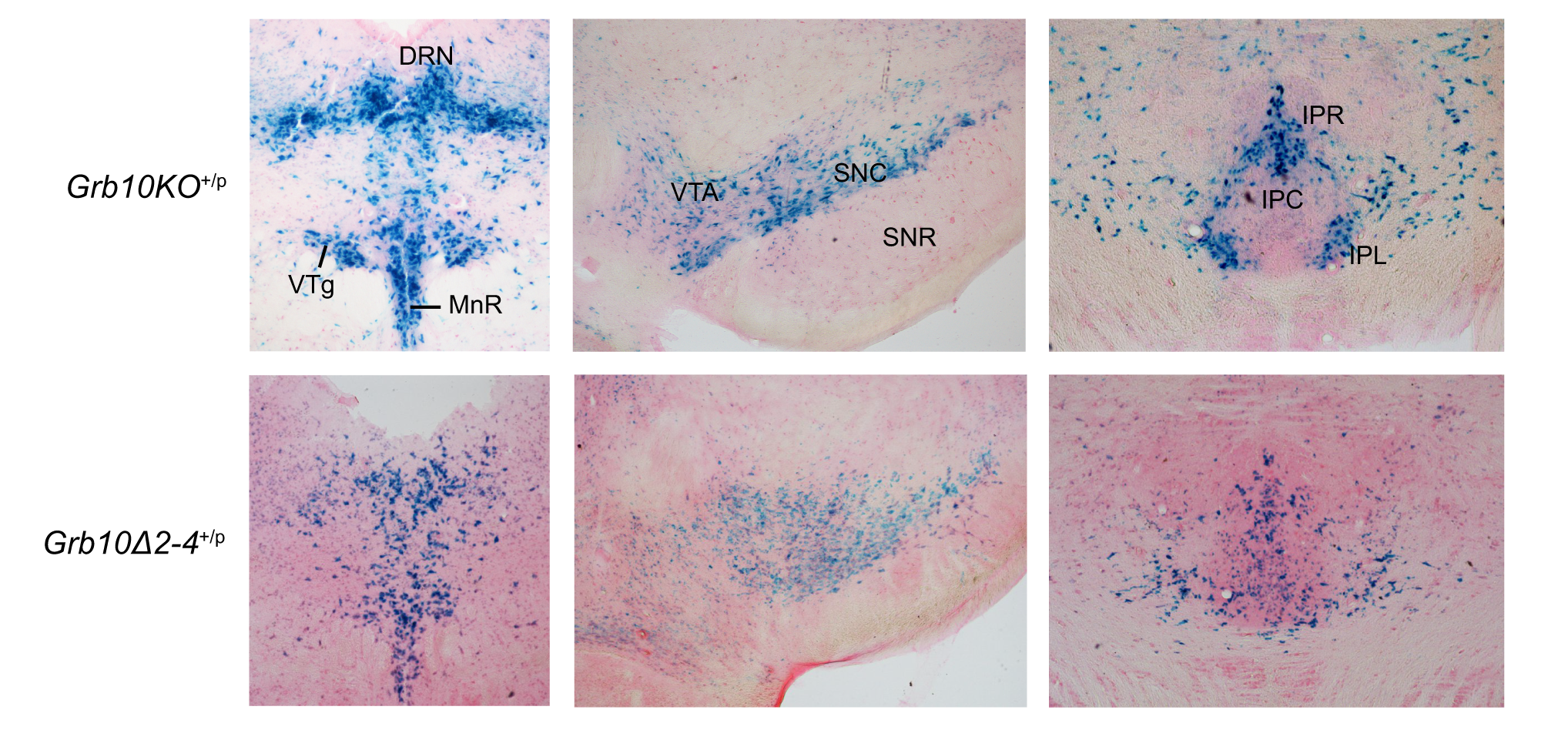

Supplement: Figure S2 — Comparative LacZ expression in Grb10KO +/p and Grb10Δ2-4 +/p adult brain sections. Adult brain sections were assayed for β-galactosidase activity for the same duration. At all sites, reporter expression was weaker in Grb10Δ2-4 +/p than Grb10KO +/p sections. (A)/(A′) Expression in the dorsal raphe nucleus (DRN), ventrotegmental nucleus (VTg), and raphe magnus nucleus (MnR). (B)/(B′) Expression in the ventrotegmental area (VTA) and the substantia nigra pars compacta (SNC) but not the substantia nigra pars reticulata (SNR). (C)/(C′) Expression in the rostral and lateral interpeduncular nucleus (IPR and IPL, respectively) but not the caudal portion (IPC). (TIF) [file pbio.1001799.s002.tif]

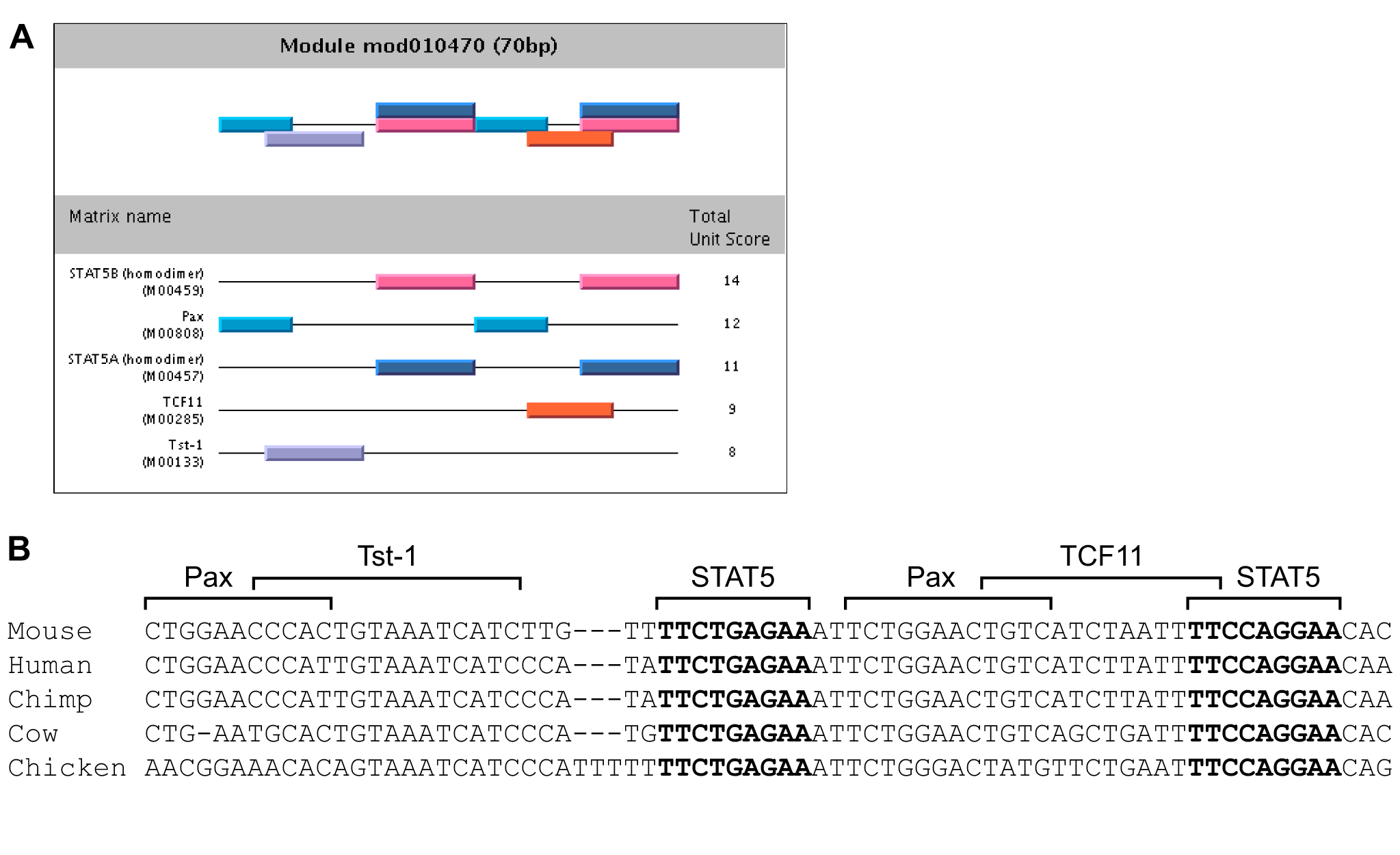

Supplement: Figure S3 — Sequence analysis of CRM1. (A) The mouse Grb10 sequence was submitted to PReMod for analysis. A single putative regulatory module of 70 bp (CRM1) was detected, consisting of a number of potential transcription factor binding sites, ranked by relative likelihood scores, calculated by an alignment with consensus sequences in the TransFac 7.2 database. TransFac module codes are presented with transcription factor names on the left hand side. (B) Sequence alignment of CRM1 between four mammalian and one avian species. Potential transcription factor binding sites identified by PReMod are illustrated with brackets. The conserved STAT5 recognition sequences are in bold text. (TIF) [file pbio.1001799.s003.tif]

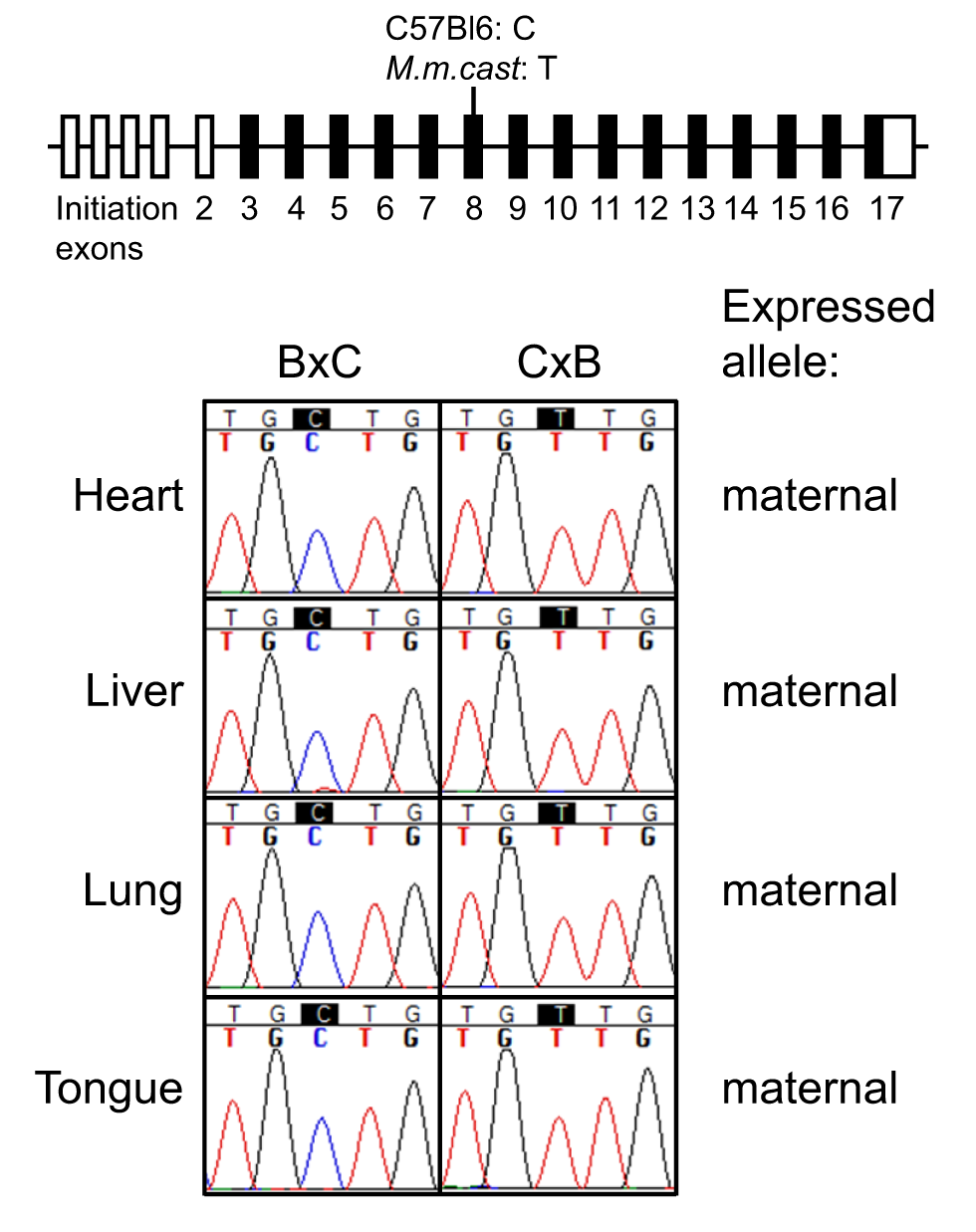

Supplement: Figure S4 — Maintenance of Grb10 imprinting in neonatal tissues. Tissues were isolated from day 1 neonates generated from intercrosses of C57Bl6 (B) and Mus musculus castaneus (C) animals. cDNA was synthesised and amplified using primers spanning a single nucleotide polymorphism (SNP) in exon 8 between the two parental strains. Amplicons were sequenced across the SNP to determine the parental origin of the expressed allele. For all tissues examined, neonates generated from a B×C cross (maternal genotype presented first) demonstrated expression exclusively from the maternally inherited allele. This finding was reproduced in tissue samples from the reciprocal cross (C×B), confirming that Grb10 is maternally expressed in postnatal heart, liver, lung, and tongue. (TIF) [file pbio.1001799.s004.tif]

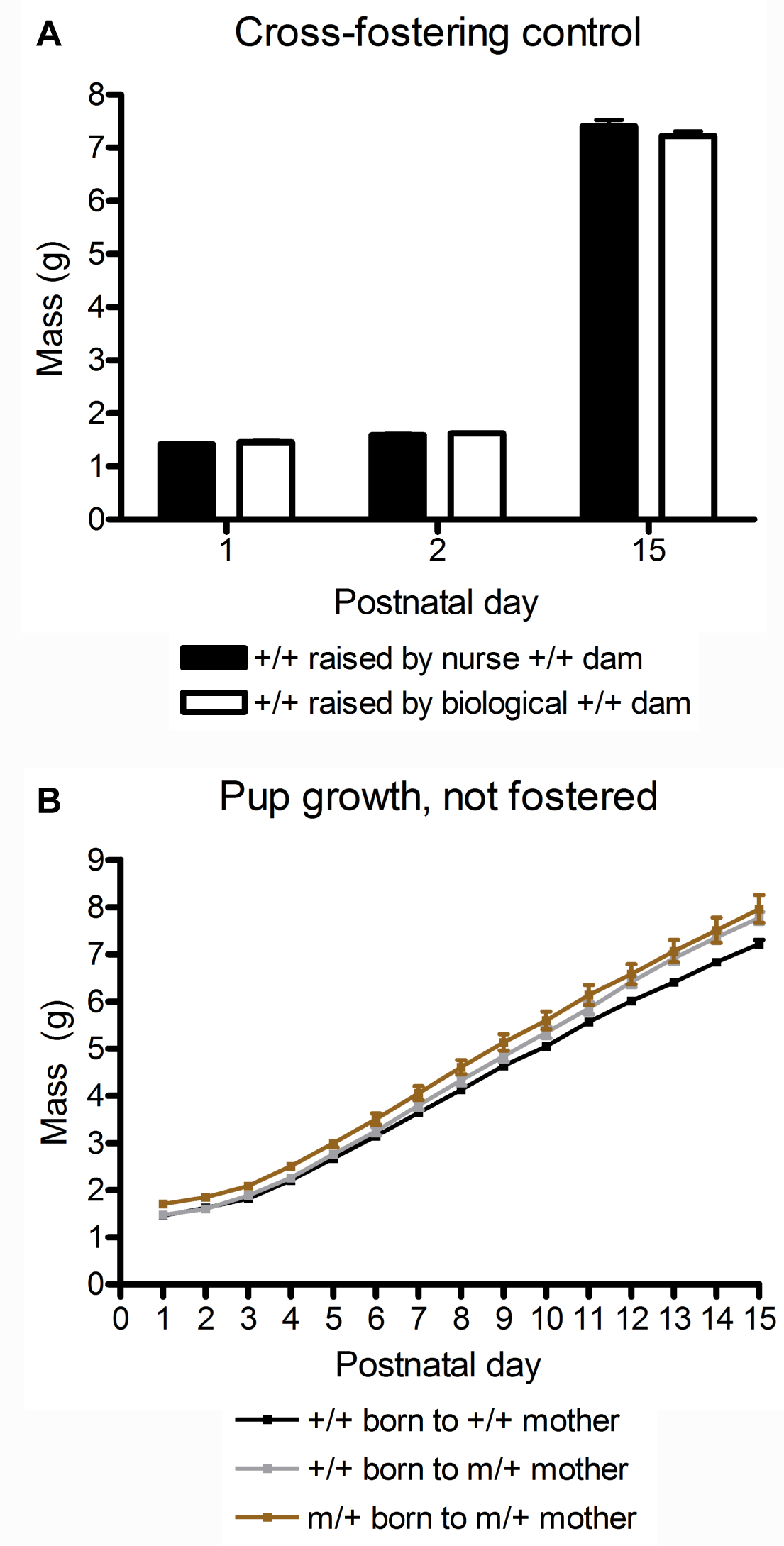

Supplement: Figure S5 — Additional postnatal growth data. (A) Growth of WT (+/+) and Grb10KO m/+ pups raised by their biological dams. The graph is reproduced from Figure 3A with the addition of the Grb10KO m/+ siblings of WT pups born to Grb10KO m/+ dams (brown line in Figure 3E), illustrating the postnatal catch-up growth of WT siblings in a mixed litter. (B) Growth of WT pups raised by biological WT and nurse WT mothers, as a control for the process of cross-fostering. No weight differences were detected on the day of birth or on the day after cross-fostering (day 2), at which point any effects of pup rejection would be detectable. No differences were detected at the end of the experimental period (day 15). These data confirm that the process of cross-fostering does not influence pup growth. Modelling of the data also confirmed no effect of cross-fostering at days 1, 8, and 15. (TIF) [file pbio.1001799.s005.tif]

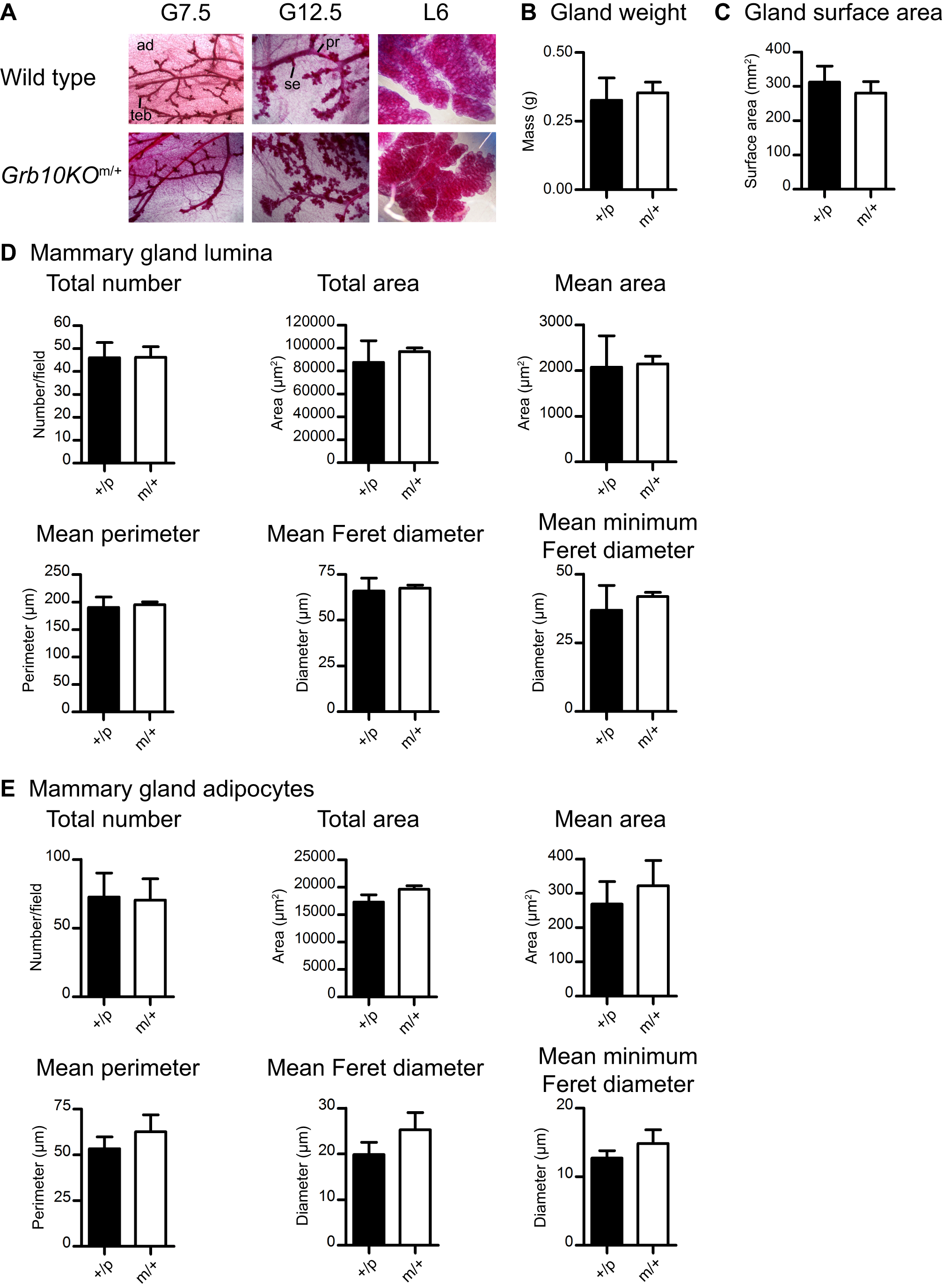

Supplement: Figure S6 — Assessment of mammary gland morphology in Grb10KO m/+ females. (A) No gross morphological differences were detected between the abdominal mammary glands of WT and Grb10KO m/+ dams at G7.5, G12.5, and L6 (WT gland images are reproduced from Figure 1C): ad, adipose tissue; teb, terminal end bud; pr, primary branch; se, secondary branch. (B–E) Abdominal mammary glands were isolated at day 5 of lactation from females raising six of their own pups (no cross-fostering). Glands from Grb10KO m/+ females were compared with those from Grb10KO +/p females, which do not show any perturbation of Grb10 expression in the mammary gland. No significant differences were found for total gland weight (B) or surface area (C), by Student's t-test. Sectioned glands were assessed for occupancy, size and shape of lumina (D) and adipocytes (E). No significant differences were found using Student's t-test. Similar experiments were also performed on glands isolated 48 hours after a forced wean, but no statistically significant differences were observed (unpublished). (TIF) [file pbio.1001799.s006.tif]

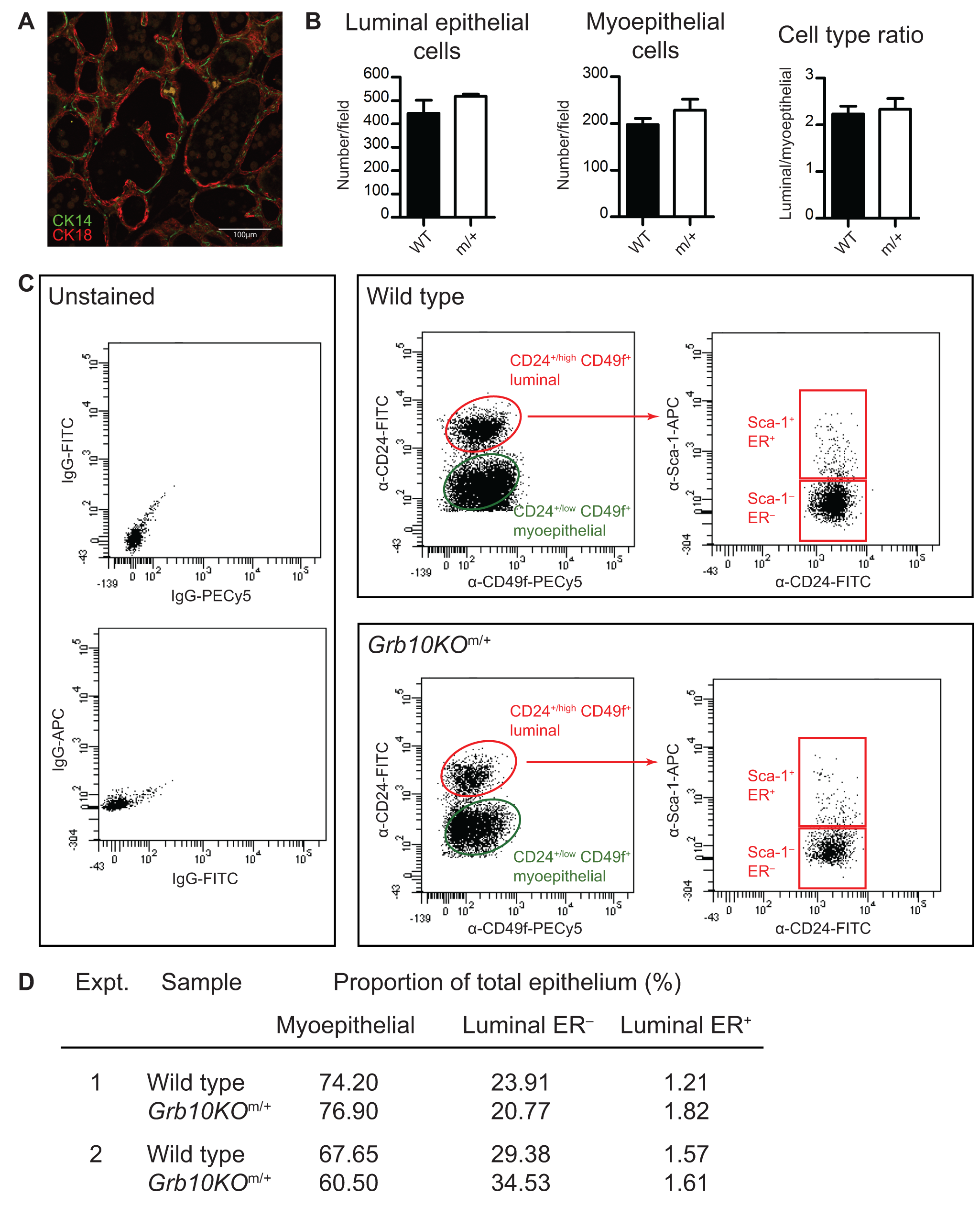

Supplement: Figure S7 — Cellular analyses of Grb10KO m/+ mammary glands. (A) Representative immunofluorescence image of a WT mammary gland section assessed for luminal epithelial and myopeithelial cell occupancy with anti-CK18 and anti-CK14, respectively. (B) No significant differences were detected between WT and Grb10KO m/+ glands for luminal epithelial or myoepithelial cell number, or for the ratio of luminal∶myopepithelial cells, assessed by Student's t-test. (C) Epithelial cell population sizes determined by FACS. Representative scatter plots of unstained control samples and samples from mid-gestation WT and Grb10KO m/+ dams stained with antibodies to CD24, Sca-1, and CD49f. Only data from the epithelial cells are shown. Non-epithelial cells were gated out prior to this analysis as described [47]. (D) Percentages of myoepithelial, luminal estrogen receptor negative (ER−), and luminal ER+ cells from two independent analyses of mid-gestation WT and Grb10KO m/+ mice as gated in (C). Note that the dot plots in (C) correspond to experiment 1. (TIF) [file pbio.1001799.s007.tif]

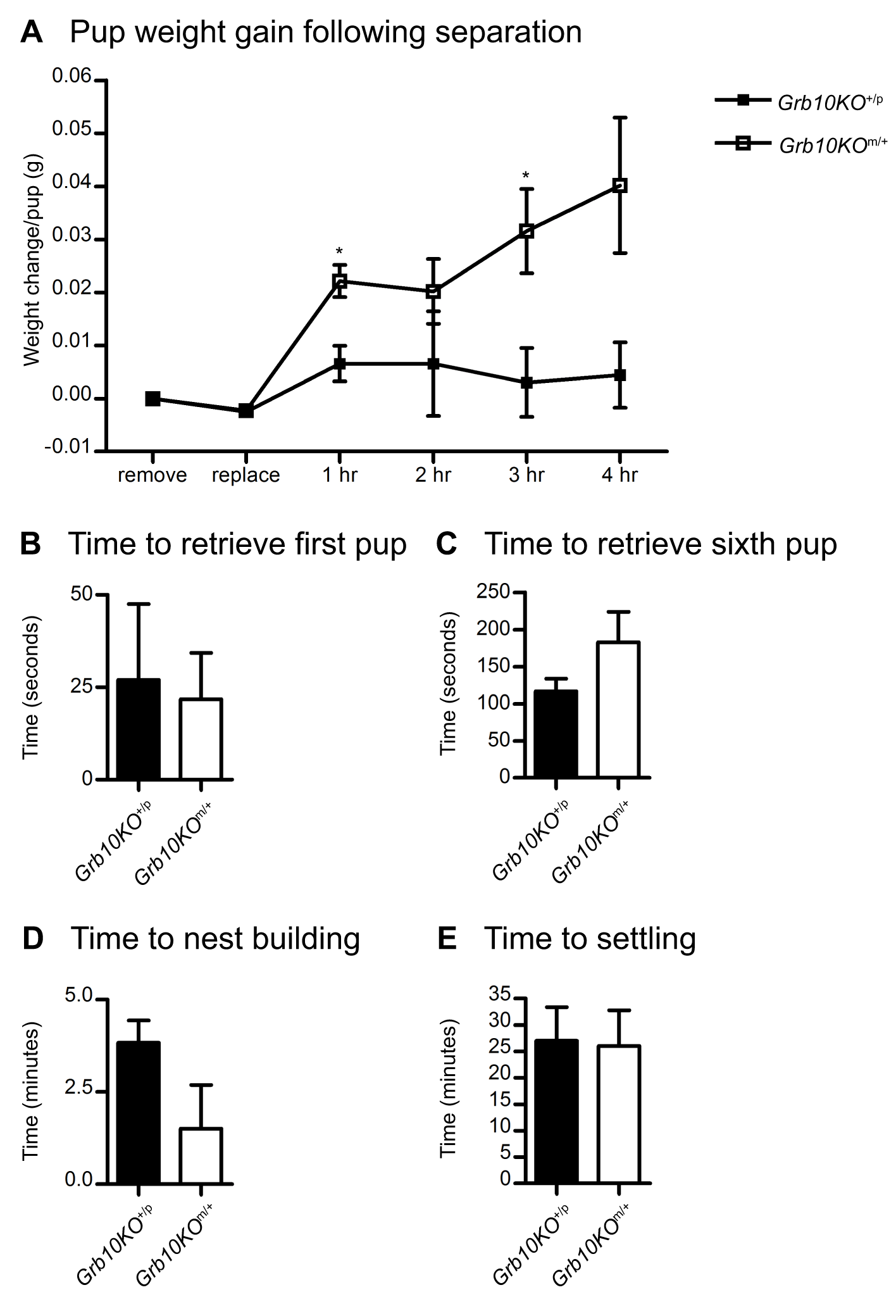

Supplement: Figure S8 — Maternal behaviour and milk letdown in Grb10KO m/+ females. Behaviour and letdown in Grb10KO m/+ dams was compared to Grb10KO +/p dams on the day of birth following separation of dam and pups for one hour. (A) Mean pup weight change following separation and reunion, as a measure of milk letdown. Letdown appeared to be greater in Grb10KO m/+ than Grb10KO +/p dams, and this was significant by Student's t-test at 1 hour and 3 hours after reunion. (B–E) Upon reunion, pups were placed in a corner of the cage away from the nest. No significant differences were observed in the time taken for the dam to retrieve the first (B) or last (C) pup, or in the time taken to begin nest building (D) or settle on the nest (E). Datasets were compared with Student's t-test. *p<0.05. (TIF) [file pbio.1001799.s008.tif]

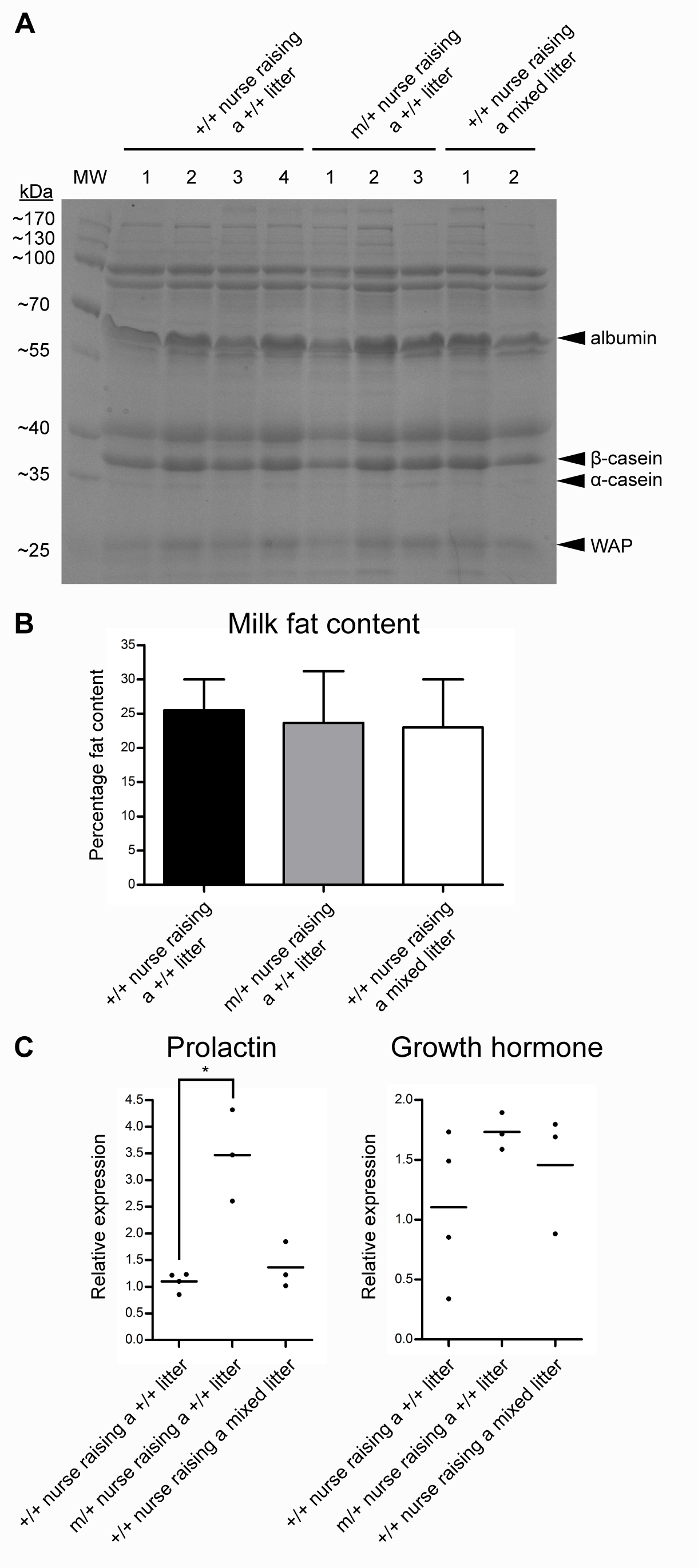

Supplement: Figure S9 — Analyses of nurses used in the cross-fostering study. (A) Milk was isolated from nurses at day 17 of lactation and analysed for protein content by SDS-PAGE. Each lane represents milk protein isolated from a separate dam. No consistent differences were observed between any of the datasets. The molecular weights (MW) of protein standards are indicated, and bands corresponding to some key milk proteins are annotated: WAP, whey acidic protein. (B) Percentage fat content of milk samples was determined. No statistically significant differences were observed between any dataset, as assessed by one-way ANOVA. Bars represent the mean ± standard error. For +/+ nurse raising a +/+ litter, n = 4; for m/+ nurse raising a +/+ litter, n = 3; for +/+ nurse raising a mixed litter, n = 2. (C) Prolactin and growth hormone expression in the pituitary glands of some nurses used in the cross-fostering study, assayed by qPCR and normalised to the expression of β-actin. Prolactin expression was elevated in the glands of Grb10KO m/+ nurses suckling WT litters, relative to WT nurse controls. No differences were observed between WT nurses raising pure WT or mixed litters. Expression of growth hormone did not differ significantly between any dataset. *p<0.05, using Kruskal-Wallis test with Dunn's multiple comparison post test. (TIF) [file pbio.1001799.s009.tif]
