# Supplementary material for: Developmental Programming Mediated by Complementary Roles of Imprinted Grb10 in Mother and Pup
Source: PLoS Biol. 2014 Feb 25;12(2):e1001799. doi: 10.1371/journal.pbio.1001799 (PMC3934836; doi:10.1371/journal.pbio.1001799)
Supplement: Table S1 — Tests of cross-fostering effect. The cross-fostering effect was tested using the same GLMM described in the Materials and Methods. The F tests all have one numerator degree of freedom, so the df column indicates denominator degrees of freedom. (DOC) [file pbio.1001799.s010.doc]

| Trait | F | df | *p* |
| --- | --- | --- | --- |
| Day 1 | 1.58 | 18.6 | 0.22 |
| Day 8 | 1.49 | 18.8 | 0.24 |
| Day 15 | 0.12 | 18.4 | 0.73 |

**Table S1. Tests of cross-fostering effect.** The cross-fostering effect was tested using the same GLMM described in the Materials and Methods. The F tests all have one numerator degree of freedom, so the df column indicates denominator degrees of freedom.
