# Supplementary material for: Developmental Programming Mediated by Complementary Roles of Imprinted Grb10 in Mother and Pup
Source: PLoS Biol. 2014 Feb 25;12(2):e1001799. doi: 10.1371/journal.pbio.1001799 (PMC3934836; doi:10.1371/journal.pbio.1001799)
Supplement: Table S2 — Pup weight modelled at days 1, 8, and 15. Pup weight was modelled as described in Materials and Methods. Num DF, numerator degrees of freedom. Den DF, denominator degrees of freedom. +/+, WT. m/+, Grb10KO m/+. Model estimated means were generated using the Least-Squares Means statement in the Mixed Procedure in SAS from the full model. (DOCX) [file pbio.1001799.s011.docx]

Day 1

| **Effect** | **Num DF** | **Den DF** | **F value** | ***p*** | **Model estimated means** | | |
| --- | --- | --- | --- | --- | --- | --- | --- |
|  |  |  |  |  | **Genotype** | **Mean** | **S.E.** |
| Pup genotype | 1 | 116 | 99.09 | < 0.0001 | +/+ | 1.42 | 0.032 |
|  |  |  |  |  | m/+ | 1.82 | 0.043 |
| Biological dam genotype | 1 | 20.9 | 0.32 | 0.5784 | +/+ | 1.64 | 0.048 |
|  |  |  |  |  | m/+ | 1.60 | 0.046 |
| Nurse-dam genotype | 1 | 19.9 | 0.53 | 0.4744 | +/+ | 1.64 | 0.046 |
|  |  |  |  |  | m/+ | 1.60 | 0.047 |
| Pup*nurse  interaction | 1 | 124 | 7.05 | 0.0090 | +/+ pup with +/+ nurse | 1.39 | 0.045 |
|  |  |  |  |  | +/+ pup with  m/+ nurse | 1.45 | 0.047 |
|  |  |  |  |  | m/+ pup with  +/+ nurse | 1.90 | 0.064 |
|  |  |  |  |  | m/+ pup with  m/+ nurse | 1.75 | 0.058 |

Day 8

| **Effect** | **Num DF** | **Den DF** | **F value** | ***p*** | **Model estimated means** | | |
| --- | --- | --- | --- | --- | --- | --- | --- |
|  |  |  |  |  | **Genotype** | **Mean** | **S.E.** |
| Pup genotype | 1 | 117 | 31.06 | <0.0001 | +/+ | 4.37 | 0.08 |
|  |  |  |  |  | m/+ | 4.90 | 0.11 |
| Biological dam genotype | 1 | 21.9 | 5.53 | 0.0281 | +/+ | 4.43 | 0.12 |
|  |  |  |  |  | m/+ | 4.83 | 0.12 |
| Nurse-dam genotype | 1 | 21 | 15.08 | 0.0009 | +/+ | 4.96 | 0.11 |
|  |  |  |  |  | m/+ | 4.31 | 0.12 |
| Pup*nurse  interaction | 1 | 126 | 15.65 | 0.0001 | +/+ pup with +/+ nurse | 4.51 | 0.12 |
|  |  |  |  |  | +/+ pup with  m/+ nurse | 4.23 | 0.12 |
|  |  |  |  |  | m/+ pup with  +/+ nurse | 5.40 | 0.16 |
|  |  |  |  |  | m/+ pup with  m/+ nurse | 4.39 | 0.14 |

Day 15

| **Effect** | **Num DF** | **Den DF** | **F value** | ***p*** | **Model estimated means** | | |
| --- | --- | --- | --- | --- | --- | --- | --- |
|  |  |  |  |  | **Genotype** | **Mean** | **S.E.** |
| Pup genotype | 1 | 123 | 15.05 | 0.0002 | +/+ | 7.60 | 0.11 |
|  |  |  |  |  | m/+ | 8.27 | 0.17 |
| Biological dam genotype | 1 | 23.1 | 21.15 | 0.0001 | +/+ | 7.40 | 0.17 |
|  |  |  |  |  | m/+ | 8.47 | 0.16 |
| Nurse-dam genotype | 1 | 21.6 | 25.60 | <0.0001 | +/+ | 8.52 | 0.16 |
|  |  |  |  |  | m/+ | 7.36 | 0.16 |
| Pup*nurse  interaction | 1 | 121 | 14.50 | 0.0002 | +/+ pup with +/+ nurse | 7.87 | 0.16 |
|  |  |  |  |  | +/+ pup with  m/+ nurse | 7.33 | 0.16 |
|  |  |  |  |  | m/+ pup with  +/+ nurse | 9.16 | 0.25 |
|  |  |  |  |  | m/+ pup with  m/+ nurse | 7.38 | 0.22 |

**Table S2. Pup weight modelled at days 1, 8 and 15.** Pup weight was modelled as described in Materials and Methods. Num DF = numerator degrees of freedom. Den DF = denominator degrees of freedom. +/+ = wild type, m/+ = *Grb10KO*^m/+^. Model estimated means were generated using the Least-Squares Means statement in the Mixed Procedure in SAS from the full model.
