# Supplementary material for: Developmental Programming Mediated by Complementary Roles of Imprinted Grb10 in Mother and Pup
Source: PLoS Biol. 2014 Feb 25;12(2):e1001799. doi: 10.1371/journal.pbio.1001799 (PMC3934836; doi:10.1371/journal.pbio.1001799)
Supplement: Table S3 — Pairwise tests of differences in pup weights at days 1, 8, and 15 as a function of the genotype of nurse and pup. Rows and columns indicate the pup/nurse genotype combinations being compared. m/+ indicates the Grb10KO m/+ genotype while +/+ indicates the WT genotype. d is the difference in means between the two categories being compared (calculated as the mean of the value indicated by the row combination minus the column combination). The estimated model means being compared are given in Table S2. (DOC) [file pbio.1001799.s012.doc]

Day 1

| Nurse→  ↓ |  | m/+ | | +/+ | |
| --- | --- | --- | --- | --- | --- |
|  | Pup→  ↓ | m/+ | +/+ | m/+ | +/+ |
| m/+ | m/+ |  | *d* = 0.30  *t* = 6.28  df = 113  *p* = <0.0001 | *d* = −0.15  *t* = −1.76  df = 43.7  *p* = 0.086 | *d* = 0.35  *t* = 4.65  df = 33.2  *p* = <0.0001 |
|  | +/+ |  |  | *d* = 0.45  *t* = 5.66  df = 35.9  *p* = <0.0001 | *d* = 0.055  *t* = 0.82  df = 19.5  *p* = 0.42 |
| +/+ | m/+ |  |  |  | *d* = 0.50  *t* = 7.96  df = 124  *p* = <0.0001 |
|  | +/+ |  |  |  |  |

Day 8

| Nurse→  ↓ |  | m/+ | | +/+ | |
| --- | --- | --- | --- | --- | --- |
|  | Pup→  ↓ | m/+ | +/+ | m/+ | +/+ |
| m/+ | m/+ |  | *d* = 0.17  *t* = 1.48  df = 115  *p* = 0.14 | *d* = −1.01  *t* = −4.78  df = 43.6  *p* = <0.0001 | *d* = −0.12  *t* = −0.63  df = 32.9  *p* = 0.53 |
|  | +/+ |  |  | *d* = 1.18  *t* = 6.00  df = 35.9  *p* = <0.0001 | *d* = −0.28  *t* = −1.71  df =20.6  *p* = 0.10 |
| +/+ | m/+ |  |  |  | *d* = 0.89  *t* = 5.96  df = 125  *p* = <0.0001 |
|  | +/+ |  |  |  |  |

Day 15

| Nurse→  ↓ |  | m/+ | | +/+ | |
| --- | --- | --- | --- | --- | --- |
|  | Pup→  ↓ | m/+ | +/+ | m/+ | +/+ |
| m/+ | m/+ |  | *d* = 0.051  *t* = 0.25  df = 119  *p* = 0.80 | *d* = −1.77  *t* = −5.47  df = 53.3  *p* = <0.0001 | *d* = −0.49  *t* = −1.77  df = 44.3  *p* = 0.084 |
|  | +/+ |  |  | *d* = 1.83  *t* = 6.21  df = 44.4  *p* = <0.0001 | *d* = −0.54  *t* = −2.38  df = 20.4  *p* = 0.027 |
| +/+ | m/+ |  |  |  | *d* = 1.28  *t* = 4.84  df =123  *p* = <0.001 |
|  | +/+ |  |  |  |  |

**Table S3. Pairwise tests of differences in pup weights at days 1, 8 and 15
as a function of the genotype of nurse and pup.** Rows and columns indicate
the pup/nurse genotype combinations being compared. m/+ indicates the
*Grb10KO*^m/+^ genotype while +/+ indicates the wild type genotype.  d is the
difference in means between the two categories being compared (calculated as
the mean of the value indicated by the row combination minus the column
combination).  The estimated model means being compared are given in Table
S2.
